# Peroxisome Proliferator-Activated Receptors (PPARs) May Mediate the Neuroactive Effects of Probiotic Metabolites: An In Silico Approach

**DOI:** 10.3390/ijms26104507

**Published:** 2025-05-09

**Authors:** Irving Parra, Alan Carrasco-Carballo, Victoria Palafox-Sanchez, Isabel Martínez-García, José Aguilera, José L. Góngora-Alfaro, Irma Isela Aranda-González, Yousef Tizabi, Liliana Mendieta

**Affiliations:** 1 Laboratorio de Neuroquímica, Facultad de Ciencias Químicas, Benemérita Universidad Autónoma de Puebla, Puebla 72592, Mexico; irving.parra@alumno.buap.mx (I.P.); mariai.martinez@correo.buap.mx (I.M.-G.); 2 Laboratorio de Elucidación y Síntesis en Química Orgánica, Facultad de Ciencias Químicas, Benemérita Universidad Autónoma de Puebla, Puebla 72592, Mexico; alan.carrascoc@correo.buap.mx; 3 Institute for Obesity Research, Instituto Tecnológico y de Estudios Superiores de Monterrey, Monterrey 64700, Mexico; victoria_palafox@tec.mx; 4 Institut de Neurociències, Universitat Autònoma de Barcelona, 08193 Barcelona, Spain; jose.aguilera@uab.cat; 5 Departamento de Neurociencias, Centro de Investigaciones Regionales “Dr. Hideyo Noguchi”, Universidad Autónoma de Yucatán, Avenida Itzáes No. 490 x 59, Mérida 97000, Mexico; galfaro@correo.uady.mx; 6 Facultad de Medicina, Universidad Autónoma de Yucatán, Avenida Itzáes No. 498 x 59 y 59A, Mérida 97000, Mexico; irma.aranda@correo.uady.mx; 7 Department of Pharmacology, Howard University College of Medicine, Washington, DC 20059, USA; ytizabi@howard.edu

**Keywords:** probiotics, metabolites, neuroprotection, neuroinflammation, gut-brain axis, gut microbiota, PPARs

## Abstract

It is well established that the gut-brain axis (GBA) is a bidirectional communication between the gut and the brain. This axis, critical in maintaining overall homeostasis, is regulated at the neuronal, endocrine, and immunological levels, all of which may be influenced by the gut microbiota (GM). Therefore, dysbiosis or disruption in the GM may have serious consequences including neuroinflammation due to overactivation of the immune system. Strategies to reestablish GM integrity via use of probiotics are being pursued as novel therapeutic intervention in a variety of central and peripheral diseases. The mechanisms leading to dysbiosis or efficacy of probiotics, however, are not fully evident. Here, we performed computational analysis on two major probiotics, namely *Lactobacillus Lacticaseibacillus rhamnosus* GG (formerly named *Lactobacillus rhamnosus*, *L. rhamnosus* GG) and *Bifidobacterium animalis* spp. *lactis (B. lactis* or *B. animalis)* to not only shed some light on their mechanism(s) of action but also to identify potential molecular targets for novel probiotics. Using the PubMed web page and BioCyc Database Collection platform we specifically analyzed proteins affected by metabolites of these bacteria. Our results indicate that peroxisome proliferator-activated receptors (PPARs), nuclear receptor proteins that are involved in regulation of inflammation are key mediators of the neuroactive effect of probiotics.

## 1. Introduction

The human gut is home to a complex and dynamic community of trillions of microorganisms, collectively referred to as the gut microbiota. When considering their genetic material, this ecosystem is termed the gut microbiome (GM). These two terms are often used interchangeably, though they represent distinct concepts. The GM, composed of bacteria, fungi, viruses, and other microorganisms, plays a critical role in maintaining physiological homeostasis, influencing processes such as nutrient metabolism, immune system regulation, and even brain function [1,2,3,4].

A balanced and healthy GM, known as *eubiosis*, is characterized by a harmonious composition of microbial species, primarily dominated by phyla such as *Firmicutes* (~64%) and *Bacteroidetes* (~23%), alongside others like *Proteobacteria*, *Actinobacteria*, and *Verrucomicrobia*. This balanced equilibrium is essential for overall health, as it supports the host’s defense against pathogens, regulates metabolic processes, and maintains the integrity of the gut barrier. In contrast, *dysbiosis* refers to an imbalance in the composition and function of the GM, often marked by a reduction in beneficial microbes and an overgrowth of opportunistic or pathogenic organisms. Dysbiosis has been linked to a wide range of health conditions, including inflammatory bowel disease, metabolic disorders, and neurodegenerative conditions such as Parkinson’s disease (PD) and Alzheimer’s disease [3,5,6,7,8,9,10,11,12,13,14,15].

The bidirectional communication between the gut and the brain, known as the gut–brain axis (GBA), is a critical pathway through which the GM influences the function of the central nervous system (CNS). GBA is regulated at neuronal, endocrine, and immunological levels, and dysbiosis can disrupt this communication, leading to neuroinflammation and other CNS-related pathologies. For instance, dysbiosis-induced activation of pro-inflammatory pathways and the production of neurotoxic metabolites can contribute to the development of neurodegenerative diseases [3,6,16,17].

To restore GM balance and counteract dysbiosis, several strategies have been explored, including fecal microbiota transplantation (FMT) [18] and the use of *probiotics* [8,19,20]. Probiotics are defined as live microorganisms that, when administered in adequate amounts (typically 10^6^ to 10^8^ colony-forming units = CFU), confer health benefits to the host. The mechanisms of action of probiotics are multifaceted and include modulation of the immune response, enhancement of gut barrier function, and production of bioactive metabolites [5,8,16,21,22,23,24,25,26,27,28,29]. One of the key mechanisms by which probiotics exert their effects is through the production of metabolites, such as short-chain fatty acids (SCFAs), tryptophan metabolites, trimethylamine-N-oxide (TMAO), bile acids, polyamines, bacterial vitamins and other bioactive compounds [3,30,31,32].

Among the most studied probiotics are *Lacticaseibacillus rhamnosus* GG (formerly named *Lactobacillus rhamnosus*, *L. rhamnosus* GG) and *Bifidobacterium animalis* spp. *lactis* (*B. lactis* or *B. animalis*), which have demonstrated efficacy in ameliorating both peripheral inflammatory conditions, such as colitis and liver diseases, as well as CNS-related neuroinflammatory conditions that may lead to neurodegeneration. For example, we have demonstrated neuroprotective and anti-inflammatory effects of bacterial probiotics, specifically *L. rhamnosus* GG and *B. lactis*, in in vivo experiments using rat models of PD [33,34]. These findings are supported by similar results from other research groups, who have reported beneficial effects of probiotics in mitigating neuroinflammation and neurodegeneration [1,22,25,26,27,35,36,37,38,39,40,41,42,43,44,45,46,47,48,49]. While these studies collectively highlight the therapeutic potential of probiotics, the precise mechanisms underlying their effects, particularly at the CNS level, remain poorly understood.

Although the roles of probiotic-derived metabolites in modulating intestinal and systemic inflammation, and metabolic processes, have been extensively studied, their specific mechanisms of action, pathways, and interactions with molecular targets in the CNS are still largely unexplored. This gap in knowledge underscores the need for further research to elucidate how probiotic metabolites exert their neuroprotective and anti-inflammatory effects in the brain, particularly through interactions with central molecular targets such as nuclear receptors. Understanding these mechanisms is crucial for developing targeted probiotic-based therapies for inflammatory and/or neurodegenerative diseases.

**Figure 1:**
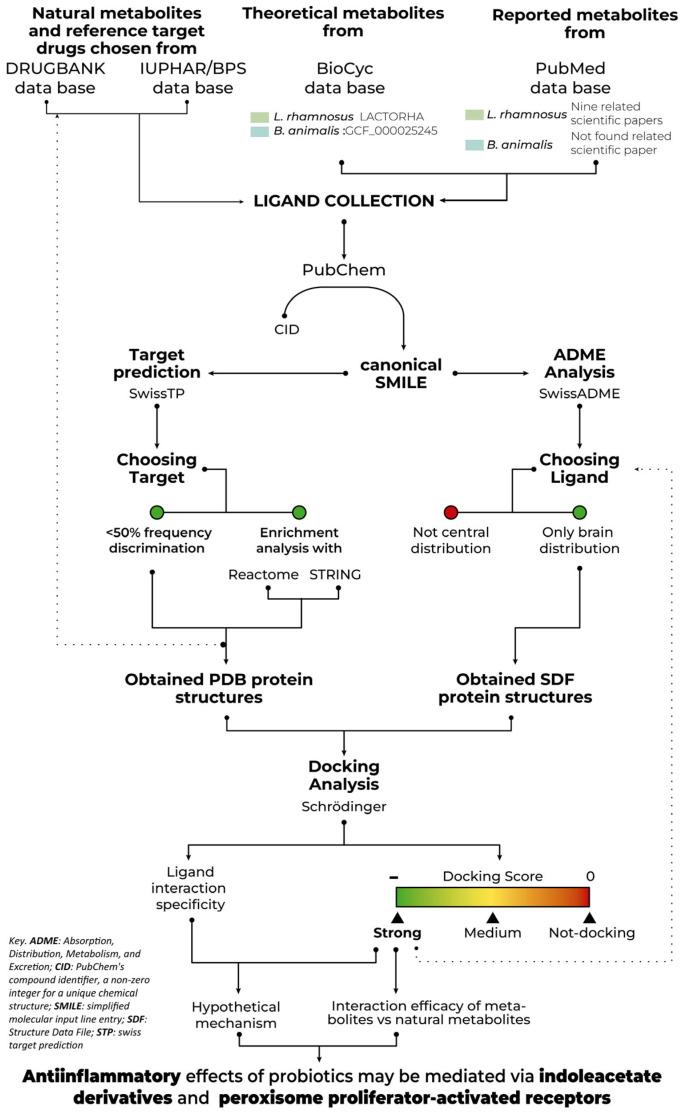
In-silico working diagram depicting theoretical and reported metabolites from *L. rhamnosus* and *B. animalis* by BioCyc Database (DB) Collection and PubMed DB. We interexchanged PubChem Compound ID list (CID) and canonical Simplified Molecular Input Line Entry Specification codes (SMILE) by PubChem platform to perform all the in-silico analysis. Targets were selected through 2D/3D target prediction, a frequency plot and an enrichment analysis. Natural metabolites, reference drugs, and protein crystals were recuperated by DrugBank Online (Academic version), International Union of Basic and Clinical Pharmacology (IUPHAR)/British Pharmacological Society (BPS) Guide to PHARMACOLOGY and Protein Data Bank (PDB), respectively. Through absorption, distribution, metabolism and excretion (ADME) theoretical analyses, only the metabolites identified in the brain were chosen. Structures of those metabolites were selected via 2D Structure Data File (SDF) for docking analysis. Based on the docking score, a second set of metabolites with a strong energy of interaction (most negative docking score) were identified. Collectively, the information extracted provided a hypothetical mechanism of action for probiotics.

Our objective in this study, therefore, was to investigate the mechanisms by which probiotic-derived metabolites exert their effects in the CNS, with a particular focus on identifying potential molecular targets, including but not limited to peroxisome proliferator-activated receptors (PPARs) and retinoid X receptors (RXRs). Our hypothesis was that the beneficial effects of probiotics, particularly *L. rhamnosus* GG and *B. lactis*, were mediated through their metabolites, which could cross the blood-brain barrier (BBB) and interact with specific target proteins in the CNS (Figure 1). We further posited that the target proteins would include regulators of inflammation, lipid metabolism, and energy homeostasis, all key players in neurodegenerative diseases.

## 2. Results

### 2.1. Structural Similarity Analysis

From the BioCyc Database (DB) Collection, 1848 metabolites were found to be formed by the bacteria *L. rhamnosus* (Assembly accession numbers: LACTORH) and *B. animalis* (GCF_000025245). From these, metabolites with five-atom, non-repeating Simplified Molecular Input Line Entry Specification codes (SMILES), with 200 characters, were selected. Using the BioCyc DB, 563 SMILES were collected from *B. animalis*, and 708 from *L. rhamnosus* (1221 metabolites in total), and using PubMed review, 266 metabolites were selected.

**Figure 2:**
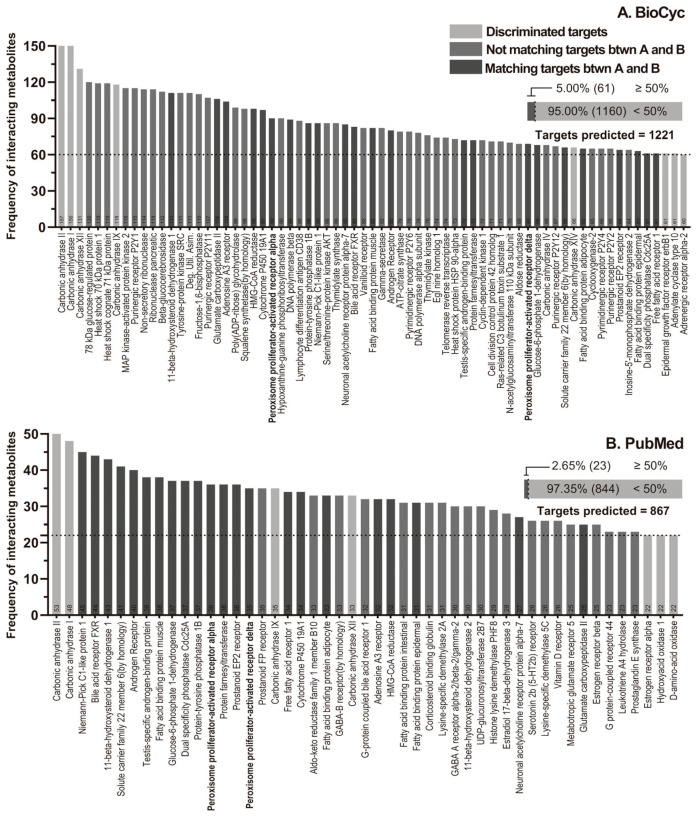
Cumulative frequency graphs. The graphs of cumulative frequencies of the target proteins are shown for the metabolites produced by the bacteria *L. rhamnosus* LGG and *B. animalis* spp. *lactis* BB12 according to the BioCyc database (**A**) and the metabolites derived from the literature review in PubMed (**B**). (**A**). STP predicted 1121 target and 1160 targets were discriminated against. (**B**). STP predicted 867 targets and 844 were discriminated against. Note that the transparency in the bars denotes that these proteins were ignored because they interacted little in the subsequent analyses. 22 similar proteins (dark gray) were identified among BioCyc and PubMed prediction models. Light gray: discriminated targets; medium gray: no matches between models; dark gray: coincidences between both models. Horizontal dot line indicates 50% of maximum frequency of interactions.

The SMILES code of each metabolite was put into the Swiss Target Prediction (STP) webpage. STP showed 1242 and 867 predicted proteins. These data were grouped according to frequency of predicted interaction of the metabolites with the proteins and were ordered from highest to lowest (Figure 2). We did not consider carbonic anhydrase enzymes (CAs; light gray) because these enzymes are mainly involved in the conversion of carbon dioxide (CO₂) to bicarbonate. Although this is considered a fundamental metabolic process, it is not directly linked to the anti-inflammatory or neuroprotective mechanisms which are the focus of this research [50,51]. Furthermore, in previous computational analyses, CAs often appear with high frequency as nonspecific binding proteins which can generate results that are not biologically relevant to the aim of our study. Finally, CAs have been shown to interact with molecules such as coumarins and other compounds in relation to the CNS and systemic diseases [52]. Proteins that were below 50% of the maximum frequency in the following analysis were excluded too (Dot line, Figure 2). The BioCyc model predicted a maximum of 61 interactions for the proteins (5% from total predicted targets) (Figure 2A), whereas PubMed model predicted about 23 interactions (2.65% from total predicted targets) (Figure 2B). Interestingly, 22 similar proteins (dark gray) were identified in both prediction models.

The full gene name approved by the Human Gene Nomenclature Committee (HGNC), gene symbol (*HGNC*:ID) and Universal Protein Resource (UniProt) code of these proteins identified consisted of the following: adenosine receptor A3 (*ADORA3*, HGNC:268), P0DMS8; aldo-keto reductase family 1 member B (*AKR1B1*, HGNC:381), P15121; androgen receptor (*AR*, HGNC:644), P10275; neuronal acetylcholine receptor subunit alpha-7 (*CHRNA7*, HGNC:1960), P36544; cytochrome P450 family 19 subfamily A member 1 (*CYP19A1*, HGNC:2594), P11511; fatty acid binding protein 3 (*FABP3*, HGNC:3557), P05413; farnesyl-diphosphate farnesyltransferase 1 (*FDFT1*, HGNC:3629), P37268; farnesyltransferase, CAAX box, subunit Alpha (*FNTA*, HGNC:3782), P49354; farnesyltransferase, CAAX box, subunit beta (*FNTB*, HGNC:3785), P49356; folate hydrolase 1 (*FOLH1*, HGNC:3788), Q04609; glucosylceramidase beta 1 (*GBA1*, HGNC:4177), P04062; 3-hydroxy-3-methylglutaryl-CoA reductase (*HMGCR*, HGNC:5006), P04035; hydroxysteroid 11-beta dehydrogenase 1 (*HSD11B1*, HGNC:5208), P28845; NPC1 like intracellular cholesterol transporter 1 (*NPC1L1*, HGNC:7898), Q9UHC9; nuclear receptor subfamily 1 group H member 4 (*NR1H4*, HGNC:7967), Q96RI1; DNA polymerase beta (*POLB*, HGNC:9174), P06746; peroxisome proliferator activated receptor alpha (*PPARA*, HGNC:9232), Q07869; peroxisome proliferator activated receptor delta (*PPARD*, HGNC:9235), Q03181; prostaglandin E2 receptor EP2 subtype (*PTGER2*, HGNC:9594), P43116; protein tyrosine phosphatase non-receptor type 1 (*PTPN1*, HGNC:9642), P18031; sex hormone binding globulin (*SHBG*, HGNC:10839), P04278; solute carrier family 22 member 6 (*SLC22A6*, HGNC:10970), Q4U2R8 (Figure 2).

### 2.2. Molecular Targets Selection

#### 2.2.1. Reactome

Enrichment analysis of metabolic pathways was conducted using UniProt codes via the Reactome web platform, with projection to the human proteome and inclusion of interactors. Pathways were filtered using a confidence interval of *p* ≤ 0.05, and disease-associated pathways were retained to elucidate the cellular, molecular, and metabolic roles of target proteins. For the BioCyc model, 61 of 62 proteins were mapped to Reactome, with 1245 pathways significantly associated with at least one protein. Similarly, 56 of 57 proteins from the PubMed model were identified in Reactome, linked to 832 pathways.

In the BioCyc model, the most statistically significant pathways included neurogenic locus notch homolog protein (NOTCH) activation and signal transmission to the nucleus, transforming growth factor beta receptor (TGF-β) signaling, nuclear receptor transcription pathways, attenuation of the heat shock transcriptional response, and nucleotide metabolism. In the PubMed model, enriched pathways were predominantly associated with nuclear receptor-mediated transcriptional regulation, including chromatin organization and control of gene expression, alongside metabolic processes such as lipid, steroid, bile acid, triglyceride, and glucocorticoid metabolism. Additionally, pathways linked to neurotransmission, notably gamma-aminobutyric acid (GABA) receptor signaling, were identified (Figure S1).

Of relevance was the pronounced involvement of lipid metabolism regulation. Pathways governing lipid biosynthesis, steroid hormone metabolism, and bile acid homeostasis were significantly enriched, underscoring their critical roles in cellular energy balance, membrane integrity, and signaling cascades. These findings highlight the interplay between nuclear receptor activity and lipid metabolic control, suggesting potential mechanisms by which target proteins modulate physiological and pathological states. The integration of these pathways signifies their collective contribution to systemic metabolic regulation as well as their broader implication in disease mechanisms and therapeutic targeting.

#### 2.2.2. Interactome

**Figure 3:**
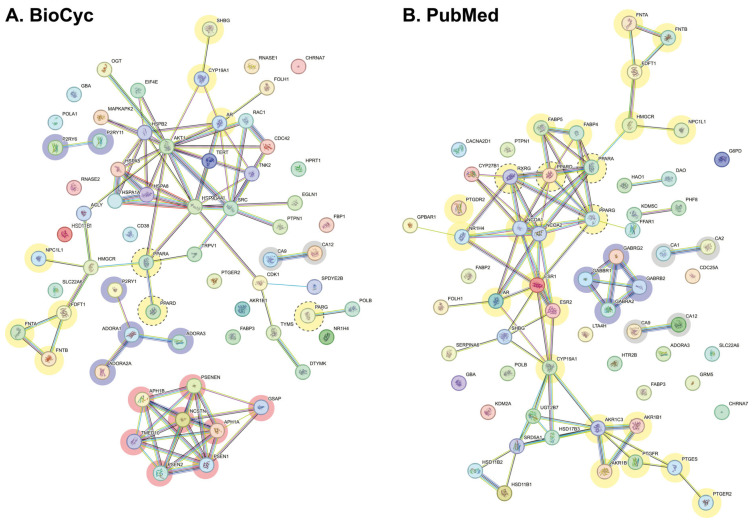
STRING Interactome. Figure shows interaction networks of the predicted target proteins for the metabolites produced by the bacteria *L. rhamnosus* LGG and *B. animalis* spp. *lactis* BB12 according to the BioCyc database (**A**), and the metabolites derived from the literature review in PubMed (**B**). Metabolites with a minimum interaction score of 0.7 and maximum of 5 are depicted. Validation model and Network Stats are shown in Supplementary Table S1. Interactions between proteins are indicated in color-equivalent categories, namely: experimentally determined (purple), neighboring genes (green), fused genes (red), co-occurrence (dark blue), co-expression (black), database (clear blue), summary of the scientific literature (text mining, yellow). Node color-coding delineates functional specialization: purple nodes denote proteins linked to neurotransmission or neuromodulation, yellow nodes emphasize lipid metabolism-associated proteins, yellow and dot line circles denote PPARs, orange nodes highlight the gamma-secretase complex, and gray nodes represent carbonic anhydrases, which exhibited no significant interactions within this network.

The protein–protein interaction network for predicted interaction metabolites produced by *L. rhamnosus* LGG and *B. animalis* subsp. *lactis* BB12 was constructed using the STRING DB, employing a stringent interaction score threshold of ≥0.7 and a maximum of five interactors (Figure 3; Supplementary Table S1). This approach ensured the inclusion of statistically robust and biologically meaningful associations. Nodes represent target proteins, while edges denote interactions, with edge colors indicating the type of experimental or computational evidence supporting each interaction (see Section 4).

The interactome integrates key proteins such as metabolic enzymes (e.g., farnesyltransferases, prostaglandin receptors), transcription regulators (e.g., nuclear receptor coactivators [NCOAs], RXR), and signaling molecules (e.g., protein kinase B [AKT], mitogen-activated protein kinases [MAPK]). The network architecture reveals distinct functional clusters, with lipid metabolism representing a dominant module. This cluster encompasses interactions among PPARs, lipid-modifying enzymes (e.g., those involved in steroid hormone synthesis and bile acid regulation), and receptors such as the androgen receptor and transient receptor potential cation channel subfamily V member 1 (TRPV1). These associations highlight a multi-tiered regulatory framework for lipid processing, spanning transcriptional control, membrane dynamics, and metabolic flux. Additional clusters are associated with inflammatory signaling (e.g., prostaglandin receptors) and transcriptional regulation (e.g., heat shock molecular chaperones, gamma-secretase subunits). Notably, enzymes linked to lipid biosynthesis and catabolism exhibit direct interactions with PPARs, underscoring their synergistic role in orchestrating lipid homeostasis highlighted as yellow nodes and dot lines. PPARs emerge as pivotal regulatory hubs, governing lipid metabolism, glucose homeostasis, and inflammatory responses. Their prominence is reinforced by diverse lines of evidence, including experimental validation, co-expression data, and literature-derived associations.

The modular organization of the interactome signifies the cooperative interplay between PPARs and ancillary proteins in mediating the systemic effects of probiotic-derived metabolites. By elucidating these interactions, the study provides a mechanistic framework for understanding host–microbe metabolic crosstalk, with implications for targeting lipid dysregulation in metabolic disorders.

Node color-coding further delineates functional specialization: purple nodes denote proteins linked to neurotransmission or neuromodulation (e.g., GABA receptors, purinergic receptors), yellow nodes emphasize lipid metabolism-associated proteins (e.g., PPARs, lipid enzymes), orange nodes highlight the gamma-secretase complex (e.g., presenilin subunits), and gray nodes represent carbonic anhydrases, which exhibited no significant interactions within the context of this network.

In the PubMed-derived interactome, PPAR-gamma (PPARG) was incorporated alongside PPAR-alpha (PPARA), PPAR-beta/delta (PPARB/D), and other lipid-associated proteins to enhance network comprehensiveness. This integration enriched the interactome, leading to expanded pathway connectivity and network growth. However, the original interactome configuration, prior to these additions, is preserved in Supplementary Figure S1. Similarly, for the BioCyc model, a subset of the interactome was spatially adjusted to optimize graphical clarity without altering biological relevance, with the original layout also archived in Supplementary Figure S1.

#### 2.2.3. Enrichment, Strength and Validation Analysis of Protein Interactions

**Figure 4:**
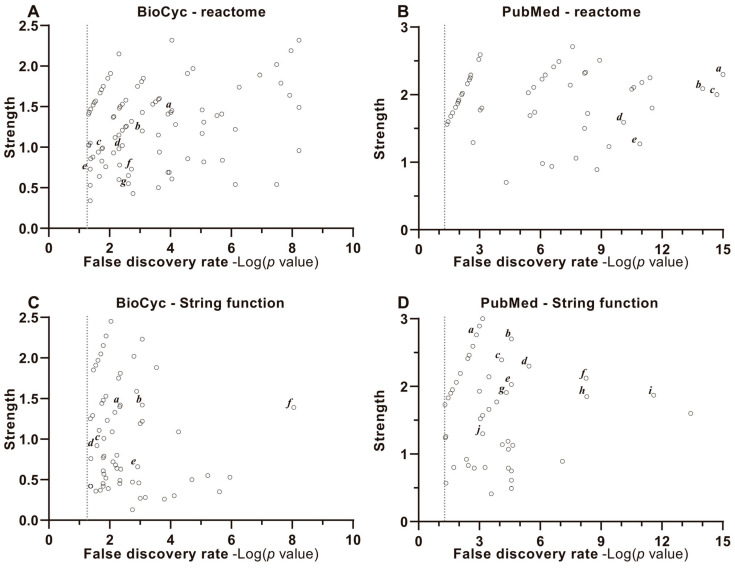
Enrichment, strength, and validation plots of protein interactions. The functional enrichment graphs of the metabolic pathways and protein–protein interaction obtained from the Reactome (**A**,**B**) and STRING (**C**,**D**) databases are shown. The *x*-axis represents Discovery Rate measured by −Log_10_(*p*-value), so the vertical dotted line represents −Log_10_(0.05) = 1.3, signifying the enrichment. Also shown are *p*-values corrected for multiple testing within each category using the Benjamini–Hochberg procedure. The *y* axis depicts the measured strength of Log_10_ (observed/expected), showing how large the enrichment effect is. It represents the ratio between (i) the number of proteins in our network that are annotated with a term and (ii) the number of proteins that are expected to be annotated with this term in a random network of the same size. Each open dot represents a protein function, pathway or cellular and molecular role of the cluster of targets analyzed. (**A**) BioCyc—Reactome. *a*. nuclear receptor transcription pathway; *b*. FCGR, a FcγR (Fc gamma receptor)-dependent phagocytosis; *c*. PPARA activating gene expression; *d*. signaling by neurotrophic tyrosine receptor kinase (NTRK); *e*. signaling by nuclear receptors; *f*. neutrophil degranulation; *g*. innate immune system. (**B**) PubMed—Reactome. *a*. nuclear receptor transcription pathway; *b*. transcriptional regulation of white adipocyte differentiation; *c*. PPARA activates gene expression; *d*. signaling by nuclear receptors; *e*. metabolism of lipids. (**C**) BioCyc—String Function. *a*. transcription coactivator binding; *b*. nuclear receptor activity; *c*. organic acid binding; *d*. carboxylic acid binding; *e*. lipid binding; *f*. Heat shock proteins (HSP) binding. (**D**) PubMed—String Function. *a*. DNA binding domain; *b*. lipid binding domain; *c*. nuclear retinoid x binding; *d*. nuclear retinoid acid receptor binding; *e*. transcription coactivator binding; *f*. nuclear receptor activity; *g*. nuclear receptor coactivator activity; *h*. transcription coregulation binding; *i*. nuclear receptor binding; *j*. transcription coactivator activity.

The functional enrichment profiles derived from the Reactome (Figure 4A,B) and STRING (Figure 4C,D) DBs are presented to elucidate the biological relevance of the identified pathways and interactions. The *x*-axis corresponds to the Discovery Rate, quantified as −Log_10_(*p*-value), with a vertical dashed line marking the significance threshold at −Log_10_(0.05) = 1.3, denoting a *p*-value ≤ 0.05. This metric reflects the statistical confidence of pathway enrichment. The *y*-axis represents Enrichment Strength, calculated as Log_10_(observed/expected), where “observed” indicates the number of target proteins annotated to a specific term within the analyzed network, and “expected” denotes the number anticipated in a randomly generated network of equivalent size. This ratio highlights the magnitude of overrepresentation for each term. All *p*-values were adjusted for multiple comparisons using the Benjamini–Hochberg procedure to control the false discovery rate (FDR).

Individual data points (open circles) correspond to specific biological functions, metabolic pathways, or molecular roles associated with the target protein clusters. Higher −Log_10_(*p*-value) values signify greater statistical significance, while elevated Log_10_(observed/expected) ratios indicate stronger enrichment effects, collectively emphasizing pathways and functions most relevant to the experimental context.

The BioCyc model proteins (Figure 4A,C) participate in cellular mechanisms related to development and are the points located in the upper or upper right part of the respective graphs. In the BioCyc model proteins (Figure 4A,C) and PubMed model (Figure 4B,D), the pathways found in the upper right are those related to the regulation of genes linked to nuclear receptors. For example, PPARs activates gene expression; assisted by coactivators, such as RXR; nuclear retinoid X binding; nuclear retinoid acid receptor binding. Interestingly, a large part of these pathways is represented in the BioCyc model and shows specificity for organic and fatty acids, organic acid binding, carboxylic acid binding and lipid binding and DNA binding domains.

It is noteworthy that the BioCyc model also includes some pathways or mechanisms related to the immune system and/or neuroprotection, such as pathways dependent on phagocytosis, neutrophil degranulation or neurotrophic receptor signaling.

### 2.3. Molecular Docking Studies

#### 2.3.1. Choice of Ligands

We used two exclusion parameters to choose key metabolites. First, we selected metabolites that could cross the BBB and, second, based on the docking data obtained, we selected the best ligand with exclusions of below percentile-95. The arbitrarily chosen 95th percentile threshold allows the identification of high-affinity interactions with the nuclear receptors.

**Figure 5:**
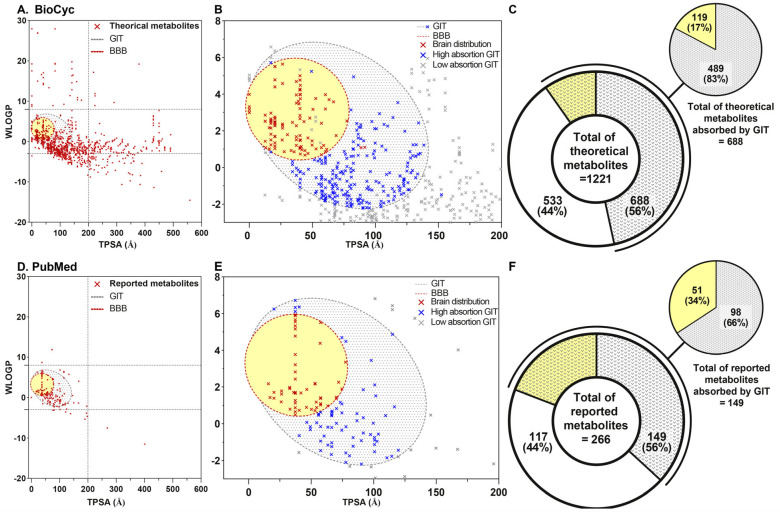
Boiled egg model for predicting the absorption of metabolites into the GIT or BBB. (**A**,**B**): molecules produced by the bacteria *L. rhamnosus* LGG and *B. animalis* spp. *lactis* BB12 according to the BioCyc database, (**D**,**E**): metabolites derived from the literature review in PubMed, and (**C**,**F**): number of metabolites absorbed by GIT and distributed to the brain. Additionally, the (**A**,**D**) plot shows all metabolites analyzed; (**B**,**E**), shows low absorption (gray dot cross), high absorption (blue dot cross) and BBB metabolites crossing (red dot cross and yellow). Epithelia of the GIT are limited by gray dotted line and BBB are limited by red dotted line. Total of theoretical metabolites (**C**) and total of reported metabolites (**F**) are shown. Metabolites of the BioCyc model (**A**–**C**) numbered 1221, of which 688 metabolites could cross GIT epithelia and 119 could cross the BBB. PubMed model metabolites (**D**–**F**) numbered 266, of which 149 metabolites could cross GIT epithelia, and 51 could cross the BBB.

We limited our study to metabolites that could cross the BBB. Applying Swiss absorption, distribution, metabolism and excretion (ADME) platform (Figure 5), it was revealed that from 1221 metabolites analyzed in the BioCyc DB, 688 (56.35%) could cross the gastrointestinal tract (GIT), and out of these, 119 (17.3%) could cross the BBB through passive diffusion. Similarly, from 266 metabolites derived from PubMed review, 149 (56%) could cross the GIT and of those, 51 (19.73%) could cross the BBB.

**Figure 6:**
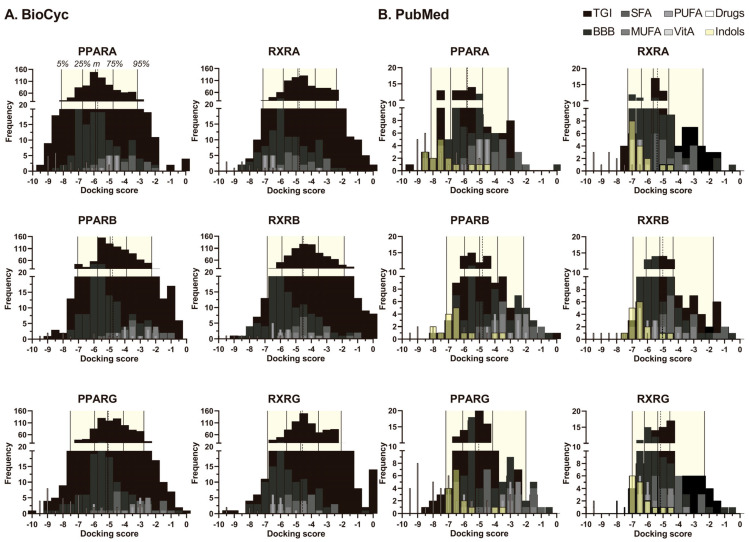
Discrimination of metabolites by frequency in the docking score. Histogram displaying the distribution of docking scores for different groups of metabolites produced by the bacteria *L. rhamnosus* LGG and *B. animalis* spp. *lactis* BB12 according to the BioCyc database (**A**) and the metabolites derived from the literature review in PubMed (**B**) across PPARs and RXRs. The metabolite categories include those capable of crossing the gastrointestinal tract (GIT, black), those that cross the blood–brain barrier (BBB, dark gray), reference molecules (SFA, MUFA, PUFA, Vitamin A; gray scale), specific drugs (white), and indole-derived metabolites (yellow). Vertical lines indicate key percentiles: 5th, 25th, median, mean, 75th, and 95th. The middle continuous line represents the median, while the middle-dotted line represents the mean. Supplementary Table S2 shows relevant data distribution around the median. Table 1 provides relevant data on score of metabolites with BBB permeability ranked above the 95th percentile.

Figure 6 and Table S2 show molecular docking histograms for the PPARA, PPARB/D, PPARG, RXR-alpha (RXRA), RXR-beta (RXRB) and RXR-gamma (RXRG) nuclear receptors versus all metabolites derived from the BioCyc DB or the PubMed review. Docking scores for 90% of metabolites ranged between −2 and −7 and median for BioCyc (PPARA: −8.125; PPARB/D: −7.047; PPARG: −7.524; RXRA: −7.138; RXRB: −6.851; RXRG: −6.842) and for PubMed (−8.12 for PPARA, −7.13 for PPARB, −7.17 for PPARG, −7.28 for RXRA, −6.94 for RXRB and −7.00 for RXRG), reflecting dataset-specific variations in ligand-receptor interactions.

The docking score distributions for PPARs and RXRs isoforms (Supplementary Figure S2 and Table S2) revealed significant differences between the BioCyc and PubMed datasets (Kruskal–Wallis, *p* < 0.001). In the BioCyc dataset, PPAR isoforms exhibited stronger docking scores compared to RXRs (*p* < 0.001). In contrast, the PubMed dataset demonstrated no significant differences between PPAR and RXR.

For BioCyc, median docking scores were in the following ranges (PPARA: −5.895; PPARB: −4.947; PPARG: −5.108; RXRA: −4.877; RXRB: −4.578; RXRG: −4.694), while PubMed showed slightly lower medians (Supplementary Figure S2 and Table S2, Figure 6), (PPARA: −5.818; PPARB: −4.984; PPARG: −5.268; RXRA: −5.632; RXRB: −5.192; RXRG: −5.34). In the BioCyc dataset, PPAR isoforms exhibited stronger median docking scores compared to RXRs (PPARA: −5.895; PPARB: −4.947; PPARG: −5.108 vs. RXRA: −4.877; RXRB: −4.578; RXRG: −4.694), with PPARA showing significantly higher binding affinity than all PPARs and RXRs isoforms (*p* < 0.001). In contrast, the PubMed dataset demonstrated no significant differences between RXRs isoforms (RXRA: −5.632; RXRB: −5.192; RXRG: −5.34), suggesting ligand–receptor interaction patterns dependent on dataset composition (Supplementary Figure S2, Figure 6); best interaction shown by the metabolites datasets was among PPARA vs. PPARB (*p* < 0.001) and PPARA vs. PPARG (*p* < 0.01).

Within the BioCyc dataset, PPARG displayed stronger binding than PPARB (*p* < 0.05) and all RXR isoforms (*p* < 0.01), while RXRA exhibited higher affinity than RXRB (*p* < 0.01) but not RXRG. In the PubMed dataset, PPARA outperformed PPAR isoforms and RXRB (*p* < 0.01). Cross-dataset comparisons do not show differences for median binding from BioCyc PPAR isoforms and PubMed PPAR, but they do for BioCyc RXR isoforms and PubMed RXR (Supplementary Figure S2).

The molecular docking analysis identified a set of metabolites with the capacity to cross the BBB and ranked above the 95th percentile in binding affinity to nuclear receptors (−8.12 for PPARA, −7.13 for PPARB, −7.17 for PPARG, −7.28 for RXRA, −6.94 for RXRB and −7.00 for RXRG). These metabolites exhibit a broad range of chemical and structural characteristics, reflecting their functional diversity and potential interactions with biological systems. Here, we provide a list and a table of these results (Table 1).

BioCyc analysis identified Indole-3-acetamide (PubChem Compound ID list, CID: 397), Phenanthrene (CID: 995), Anthraquinone (CID: 6780), P-menthan-3-one (CID: 6986), Phenothiazine (CID: 7108), Alpha-phellandrene (CID: 7460), Benzoin (CID: 8400), Beta-phellandrene (CID: 11142), 3-carene (CID: 26049), Stilbene oxide (CID: 28649), Menthone lactone (CID: 62349), 3-chlorobenzyl alcohol (CID: 70117), Pinoresinol (CID: 234817), Maackiain (CID: 363863), 4′-o-methylisoflavone (CID: 439901), Retinol; vitamin A (CID: 445354), Nsc636229 (CID: 494912), Medicarpin(p) (CID: 623060), Ibuprofen anion (CID: 3326923), 1-phenylpropan-2-ylazanium (CID: 4055279), Hinokiresinol (CID: 5377291), (z)-hinokiresinol (CID: 6440617), 4′-hydroxyisoflavone (CID: 10966551), Cis-12,13-epoxy-9-octadecenoic acid (CID: 25246088), Noroxomaritidine (CID: 77916059), Oxomaritinamine (CID: 146037227). Meanwhile, PubMed metabolites included 4-trans-4-Hydroxycinnamic acid (CID: 322), 2-(1H-indol-3-yl)acetaldehyde (CID: 800), Indole-3-pyruvic acid (CID: 803), Tryptamine (CID: 1150), 3-Indolepropionic acid (CID: 3744), 3-(4-Hydroxyphenyl)propionic acid (CID: 10394), Tryptophol (CID: 10685), Indol-3-acrylic acid (CID: 14558), 3-Indoleglyoxylic acid (CID: 73863).

Among the most relevant compounds, indole-3-acetamide (CID: 397) was identified as an indole derivative with significant interactions with PPARA and RXR subtypes, highlighting its potential role in modulating nuclear receptor activity. Other notable compounds include phenanthrene (CID: 995) and anthraquinone (CID: 6780), both of which are polycyclic aromatic hydrocarbons with structural features that allow interactions with PPARG and RXRs. Phenothiazine (CID: 7108) is a heterocyclic compound, with strong binding affinity to PPARGs and RXRs subtypes, suggesting potential anti-inflammatory properties.

Terpenoid compounds such as p-menthan-3-one (CID: 6986), α-phellandrene (CID: 7460), and β-phellandrene (CID: 11142) were identified with notable interactions with RXRs, indicating their relevance in metabolic regulation. Additionally, flavonoid and isoflavone derivatives, including pinoresinol (CID: 234817), maackiain (CID: 363863), and 4′-o-methylisoflavone (CID: 439901), demonstrated significant binding to PPARG, reinforcing their role as potential modulators of nuclear receptor activity. Lipophilic compounds such as retinol (CID: 445354) and cis-12,13-epoxy-9-octadecenoic acid (CID: 25246088) exhibited strong interactions with both PPARG and RXRs, supporting their involvement in lipid metabolism and anti-inflammatory pathways. Notably, ibuprofen anion (CID: 3326923) showed high-affinity binding to PPARA, indicating its potential role in modulating inflammatory responses through nuclear receptor activation. Additionally, a subset of indole and phenylpropanoid derivatives was identified with high binding affinities, further highlighting the relevance of microbiota-derived metabolites in nuclear receptor modulation. These include 4-trans-4-hydroxycinnamic acid (CID: 322), 2-(1H-indol-3-yl)acetaldehyde (CID: 800), indole-3-pyruvic acid (CID: 803), tryptamine (CID: 1150), 3-indolepropionic acid (CID: 3744), 3-(4-hydroxyphenyl)propionic acid (CID: 10394), tryptophol (CID: 10685), indol-3-acrylic acid (CID: 14558), and 3-indoleglyoxylic acid (CID: 73863). These metabolites, derived from microbial metabolism of tryptophan and phenylpropanoids, suggest potential neuroactive and anti-inflammatory roles through their interactions with PPARs and RXRs.

Interestingly, indole (CID: 798) and 2-(1H-indol-3-yl)acetate (CID: 801) were found in both theoretical BioCyc data and PubMed-reviewed metabolome data, suggesting that these metabolites are actively produced by *L. rhamnosus* but not by *B. lactis*. Furthermore, their presence in metabolomic studies suggests an increase in production following microbial metabolism. In contrast, 2-(1H-indol-3-yl)acetamide (CID: 397) was only identified in BioCyc, indicating that *L. rhamnosus* theoretically produces this metabolite, though it was not detected in experimental metabolome data. This suggests that *L. rhamnosus* might naturally convert indolacetamide to indolacetate through an amidase (EC 3.5.1.4). Additionally, several indole-derived metabolites were exclusively found in PubMed-reviewed data but not in BioCyc, implying that their production could result from metabolic activity of gut microbiota species such as *Bacteroides*, *Bifidobacterium*, *Clostridium*, *Peptostreptococcus*, *Lactobacillus*, and *Enterobacteria*. This finding highlights the intricate metabolic interplay within the gut microbiome in vivo [1].

Docking score analyses (two-way ANOVA) revealed significant differences in binding affinities between PPARs and RXRs receptor isoforms across clusters of natural metabolites, with pronounced variations among long-chain fatty acids (LCFAs), monounsaturated fatty acids (MUFAs), and polyunsaturated fatty acids (PUFAs) (Supplementary Figure S2). For LCFAs, PPARB exhibited significantly distinct docking profiles compared to RXRA and RXRG (*p* < 0.001), as did PPARG versus RXRA and RXRG (PPARG/RXRA and PPARG/RXRG) (*p* < 0.001), with moderate differences observed between PPARB/RXRB and PPARG/RXRB (*p* ≤ 0.05). In the MUFA cluster, robust disparities were observed between PPARB and all RXR subtypes (RXRA, RXRB, RXRG: *p* < 0.001), as well as PPARG versus RXRA and RXRG (*p* < 0.001). Additional contrasts included PPARA/RXRA (*p* ≤ 0.05), PPARA/RXRG (*p* ≤ 0.01), and PPARG/RXRB (*p* ≤ 0.05). Among PUFAs, PPARA diverged significantly from PPARB and RXRA (*p* ≤ 0.05), while PPARB and PPARG displayed marked differences relative to all RXR subtypes (*p* < 0.001).

Comparative analyses between natural fatty acids and indole metabolites identified distinct ligand–receptor interaction profiles. Indole metabolites demonstrated superior binding affinities (more negative docking scores) across PPARA, PPARB, and PPARG compared to SCFAs, medium-chain (MCFAs), LCFAs, MUFAs, and PUFAs (*p* ≤ 0.05 for most comparisons; *p* < 0.001 for key contrasts). Specifically, PUFAs and indole metabolites exhibited enhanced PPARA binding over SCFAs, LCFAs; greater PPARB affinity relative to LCFAs and PUFAs; and stronger PPARG interactions compared to LCFAs and MUFAs. Similarly, within RXR isoforms, PUFAs and indole metabolites outperformed SCFAs and LCFAs (RXRA, RXRB, RXRG: *p* ≤ 0.05 or *p* < 0.001), with additional advantages over MUFAs in RXRA and RXRB (*p* ≤ 0.05).

Ketone bodies (Kb) and retinoids were excluded from statistical analyses due to limited sample sizes. However, exploratory observations indicated that SCFAs and Kb exhibited weaker binding across PPAR/RXR subtypes compared to reference drugs, which showed the highest affinities. Notably, retinoids, including retinol (CID: 445354) and retinoic acid (CID: 449171), displayed strong interactions with RXRs, consistent with their roles as nuclear receptor ligands. While indole metabolites demonstrated more negative docking scores than several fatty acids, their affinities remained comparable to eicosapentaenoic acid (EPA) and docosahexaenoic acid (DHA) (Figure 6, Table 2) but were surpassed by vitamin A metabolites.

Of note: indole metabolites, which are generally distributed to the brain, have a neurobiological relevance because of their binding potentials, which exceed that of the endogenous fatty acids. This finding suggests a mechanistic basis for the CNS effects associated with *L. rhamnosus* probiotics, positioning indole derivatives as potential modulators of cerebral PPAR/RXR signaling pathways.

#### 2.3.2. Molecular Interaction and Multiple Sequence Alignment

The integration of data from drug molecules and the chemical classification of natural ligands has established a robust comparative framework, enabling the evaluation of reference drugs used in clinical settings, natural ligands, and potential compounds. The observation of highly negative docking scores, coupled with interactions involving similar amino acid residues, supports the hypothesis that the binding affinities of certain metabolites may hold clinical relevance. These findings underscore the utility of molecular docking models in predicting the biological activity and potential efficacy of novel compounds. Furthermore, the identification of metabolites exhibiting interaction profiles comparable to those of reference drugs suggests that such compounds may play endogenous modulatory roles in receptor signaling pathways or serve as a foundation for the design of new molecules with optimized pharmacological properties.

**Figure 7:**
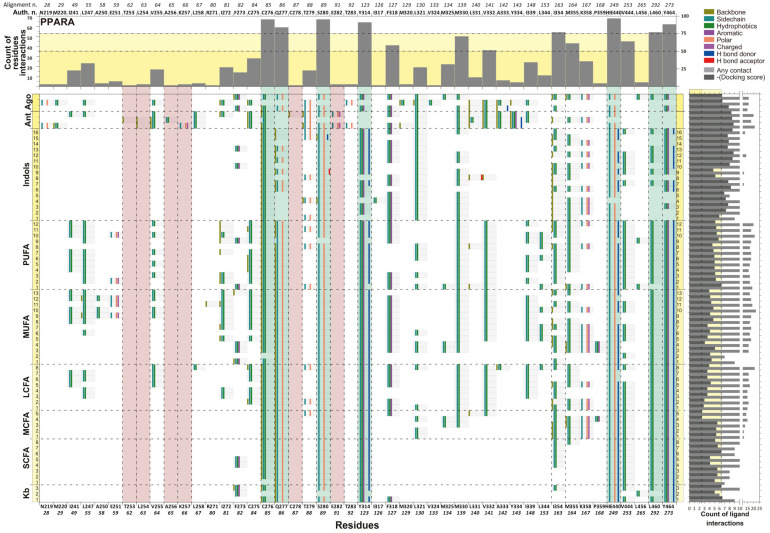
Interaction map or interaction fingerprint of PPARA nuclear protein. The map of interactions between nuclear receptor residues (horizontal axis) and natural metabolites, drug reference and indole metabolites are shown (vertical axis). The upper quadrant shows the frequency of any contact (light gray) of the residues with respect to the metabolites, and the yellow panel and dotted line indicate specific residues that interact with ≤50% and ≤75% of total metabolites, respectively. The number of aminoacidic residues are according to alignment and author crystal numbering. The right quadrant shows the frequency of any interactions of the metabolites, with respect to amino acid residues (light gray) and the negative of docking score (dark gray), and the dotted line and yellow panel indicate the maximum and minimum docking score of reference metabolites, respectively. The main window shows the specific interaction of each amino acid residue and the type of interaction with each of the metabolites. Horizontal dots indicate the limit of different metabolites or drugs and are labelled by a number relevant to the name in Table 4 and Figure S5. Vertical dots indicate specific aminoacidic residues. Vertical green squares indicate specific aminoacidic residues that interact with >75% of metabolites. Vertical red squares indicate a specific aminoacidic residue that interacts with only an antagonist. The numbers of metabolite interactions are color-coded as follows: gray: any interaction; gold: with skeleton; turquoise: with side chain; flag green: with hydrophobic residues; purple: with residues that have aromatic rings; orange: with polar residues; magenta: with charged polar residues; blue: with hydrogen bond donors; red: with hydrogen post acceptors; and white: no interaction. Kb: ketone body; SCFA: short chain fatty acid; MCFA: medium chain FA; LCFA: long chain FA; MUFA: mono-unsaturated FA; PUFA: poly-unsaturated FA; Ant: antagonist; Ago: agonist.

Figure 7 and Figure S3 illustrate the molecular interactions, specifically ligand atoms positioned within the required distance of receptor atoms, along with interaction maps between nuclear receptor residues. The horizontal axis numbering is based on a consensus derived from multiple sequence alignments (Supplementary Figure S4), drug molecules (Table 3), reference natural compounds, and indole metabolites. Figure S4 highlights the alignment window, where conserved regions are color-coded by Taylor et al. [53]. Notably, nuclear receptors exhibit a high degree of conservation, particularly within the ligand-binding region (Supplementary Figure S4).

Our results reveal that PPARs interact with ligands primarily through key residues. Specifically, PPARA interacts with the ligand through residues N219, M220, I241, L247, A250, E251, T253, L254, V255, A256, K257, L258, R271, I272, F273, C275, C276, Q277, C278, T279, S280, E282, T283, Y314, I317, F318, M320, L321, V324, M325, M330, L331, V332, A333, Y334, I339, L344, I354, M355, K358, P359, protonated histidine (HIE)440, V444, L456, L460, and Y464. Similarly, PPARB interacts through residues M228, I249, L255, W264, V281, F282, R284, C285, Q286, C287, T288, T289, E291, T292, E295, H323, I326, F327, L330, I333, V334, L339, L340, V341, A342, N343, G344, V348, F352, L353, I363, I364, K367, F368, HIE449, M453, L469, and Y473. Likewise, PPARG interacts via residues F226, P227, L228, I249, L255, Q259, F264, HIE266, A278, R280, I281, F282, G284, C285, Q286, R288, S289, A292, E295, I296, HIE323, I326, Y327, M329, L330, L333, V339, L340, I341, S342, E343, G344, M348, L353, L356, F360, F363, M364, HIE449, and L453. The upper quadrant of Figure 7 and Supplementary Figure S3 illustrates the frequency of any contact (light gray) between these residues and metabolites.

As shown in Figure 7, Figures S3A,B and S4A, and Table 4A, PPAR nuclear receptors have 13 (PPARA), 12 (PPARB), and 7 (PPARG) residues that interact with more than 50% of the analyzed ligands. Furthermore, 8 (PPARA), 3 (PPARB), and 2 (PPARG) residues interact with more than 75% of the analyzed ligands. Although PPARs exhibit approximately 60% sequence overlap, the degree of similarity in ligand interactions varies. For residues interacting with more than 50% of ligands, PPARA and PPARB share an 85% coincidence rate, while PPARA and PPARG share only 33%. For residues interacting with more than 75% of ligands, the coincidence rate is 37.5% for PPARA vs. PPARB and 12.5% for PPARA vs. PPARG. Residue number 85, based on consensus numbering (corresponding to PPARA: C275, PPARB: C285, PPARG: C285), is highly conserved across all three receptors (Table 4A).

The ligand-binding domain (LBD) is composed of Arm I, Arm II, the entrance region, the charge clamp, and key structural domains such as the Activation function-2 helix (AF-2 helix), which is essential for transcriptional activation and the binding of antagonists that prevent coactivator recruitment [54]. According to our findings, residues interacting with more than 75% of ligands, including C276, Q277, S280, Y314, HIE440, and Y464 in PPARA, belong to Arm I. Similarly, C285, T289, and HIE449 in PPARB are part of Arm I, while L469 is within Arm II, and E471 and K301 contribute to the charge clamp. In PPARG, C85 and R288 are localized in Arm I. Residues interacting with more than 50% of ligands, such as F318, M330, and V332, are part of Arm I, while E251, T253, L254, M355, A256, K257, L258, I339, and V444 are components of Arm II in PPARA. In PPARB, Q286, F327, L330, V341, H323, and Y473 are associated with Arm I, whereas I363, I364, and M453 belong to Arm II. For PPARG, residues Y327, L330, L341, S289, L326, HIE449, and I341 are located in Arm I (Supplementary Table S3).

Residues interacting with less than 50% of ligands, such as K358, I241, L247, A250, V255, R271, I272, C275, C278, T279, T283, I317, M320, L321, V324, M325, L331, A333, Y334, L344, P359, and L456, belong to Arm II, while N219 and M220 form part of the entrance region in PPARA. In PPARB, residues F352, F282, M228, I249, L255, W264, V281, R284, C287, T288, E291, T292, E295, I326, I333, V334, L339, L340, A342, N343, G344, V348, L353, K367, and F368 contribute to Arm II. Similarly, in PPARG, residues F363 and L453 belong to Arm I, while F360, F264, F226, P227, L228, I249, L255, Q259, HIE266, A278, R280, I281, F282, G284, Q286, A292, E295, I296, HIE323, M329, V339, L340, S342, E343, G344, M348, L353, L356, and M364 constitute Arm II (Supplementary Table S3).

Additionally, we identified key residues involved in agonist binding: PPARA: C276, Q277, S280, Y314, F318, M330, V332, I354, K358, H440, V444, L460, and Y464; PPARB: C285, Q286, T289, H323, F327, L330, V341, I363, I364, H449, M453, L469, and Y473; and PPARG: C285, R288, S289, L326, Y327, L330, and L341. Specific residues associated with antagonist binding include T253, L254, A256, K257, C278, and E282 in PPARA; C287, E291, and E295 in PPARB; and R288, S289, and E295 in PPARG. Finally, we identified residues that bind ligands uniquely to each PPAR subtype: M220, E251, T253, L254, A256, L258, and L456 in PPARA; F352 in PPARB; and F226, A278, I296, L356, and F360 in PPARG (Table 4).

Curiously, the antagonist drug interacts with both PPARA and PPARB on residue 87 (PPARA: C278 and PPARB: C287), and residue 91 (PPARA: E282 and PPARB: E291). However, PPARG conserves Glu on the same consensus numbering, and does not appear as dock residue. Hence, it is possible that this residue is responsible for the antagonist effect (Table 4A).

The aminoacidic residues that interact with the indole metabolites are likely agonist ligands. Most indole metabolites have a more negative docking score than natural ligands. Here, the interaction is stronger for PPARA than the rest of the PPAR proteins.

Figures S2C–E and S3B and Table 4B show that RXR nuclear receptors have respectively 13 (RXRA), 12 (RXRB) and 1 (RXRG) residues that interact with >50% of ligands analyzed, and 6 (RXRA), 6 (RXRB) and 7 (RXRG) residues that interact with >75% of ligands analyzed. RXRs isoforms show ~90% identity and a percentage of coincidence of 85% (RXRA vs. RXRB), 80% (RXRA vs. RXRG), and 92% (RXRB vs. RXRG) on residues that interact with >50% of ligands analyzed. On the other hand, RXRs have a co-identity of 39% (RXRA vs. RXRB), 46% (RXRA vs. RXRG), and 50% (RXRB vs. RXRG) with residues that interact with >75% of analyzed ligands. Residue number 50 (on author numbering RXRA: I268, RXRB: I339, RXRG: I269), residue number 95 (on author numbering RXRA: F313, RXRB: F384, RXRG: F314), residue number 127 (on author numbering RXRA: I345, RXRB: I416, RXRG: I346), residue number 214 (on author numbering RXRA: C432, RXRB: C503, RXRG: C433), and residue number 218 (on author numbering RXRA: L436, RXRB: L507, RXRG: L437) are all conserved residues with possible mediation of the antagonist effects (Table 4B).

Thus, aminoacidic residues that interact with indole-derived metabolites are likely agonist ligands. Interestingly, vitamin A residues have a more negative docking score than indole-derived metabolites. However, 2-(1H-indol-3-yl)acetaldehyde (CID: 800) has the same docking score as retinal (CID: 638015) on RXRB protein.

## 3. Discussion

Human and animal guts are colonized by microbiota, a cluster of bacteria, archaea, fungi, and viruses, which coexist in symbiosis with their host. This complex ecosystem of microorganisms (GM) plays a critical role in maintaining overall homeostasis, including that of the immune system. Moreover, GM, through its bidirectional communication with the brain, forms the GBA, which is essential in ensuring normal brain functions. Dysbiosis or disruption of this axis could result in various neuropsychiatric and/or neurodegenerative diseases. To this end, use of probiotics to counter dysbiosis has gained significant ground in recent years. The exact mechanism of action of probiotics, however, remains unknown. It is suspected that at least some of the effects are mediated via the production of bioactive metabolites. This contention is supported by the findings that metabolites such as SCFAs are key in modulating immune and metabolic functions. The probiotics most used in dietary supplements or any pharmaceutical product are likely to contain *Lactobacillus* and *Bifidobacterium*, and indeed, several strains such as *L. rhamnosus* and *B. lactis* have demonstrated beneficial attributes in clinical and preclinical studies [1,3,5,22,25,26,27,33,34,35,36,37,38,39,40,41,42,43,44,45,46,47,48,49,55,56,57,58,59].

Identification of active metabolites and their mechanism of action may not only provide a better understanding of probiotic effects but can also identify novel therapeutic targets [60]. Although the role of probiotic-derived metabolites in regulating intestinal and systemic inflammation as well as metabolic processes has been widely investigated, their specific mechanisms of action, the pathways involved, and their interactions with molecular targets in the central nervous system remain largely unknown. This lack of knowledge highlights the need for further studies to elucidate how these probiotic metabolites exert their neuroprotective and anti-inflammatory effects in the brain, especially through their interaction with nuclear receptors and other central targets. Understanding these mechanisms is critical for the development of probiotic therapies targeting neurodegenerative and inflammatory diseases.

In this regard, using state of the art in silico analysis, we have identified the nuclear receptor proteins, PPARs and indole-derived metabolites as key mediators of the neuroactive effects of two commonly used probiotics. Below, we specifically discuss other pertinent findings.

Our analysis indicates that the target proteins identified had a significant role in metabolism of lipids, binding to the carboxylic group, and affecting transcription factors (Figure 2, Figure 3 and Figure 4 and Figure S1). PPARs are nuclear receptors (NRs) that control many cellular and metabolic processes. PPARs require heterodimerization with RXR-alpha (RXRA), RXR-beta (RXRB), or RXR-gamma (RXRG) to bind to DNA response elements in the promoters and enhancers of target genes. Together, they interact with nuclear receptor coactivators (NCOA1 and others) to promote expression of target genes in the presence of agonists or other activating signals [61]. These proteins are ligand-activated transcription factors with three identified isotypes—PPARA, PPARB/D and PPARG—in mammals. These isotypes display differential tissue distribution and specific functions (Figure S5).

Therefore, PPAR receptors may be an interesting target to be studied in metabolite-protein interaction models derived from the microbiota (Figure S5) [62,63,64,65,66].

Our molecular docking results demonstrated that indole metabolites have stronger interactions with PPARs compared to Kb, SCFA, MCFA and LCFA (Table 2, Supplementary Figure S2). This finding is significant as it suggests a new class of compounds that could modulate PPARs activity more effectively.

Indole-derived metabolites could be derived from tryptophan in the diet, the gut microbiota life cycle and, according to our data, also from the probiotic bacterium *L. rhamnosus* (but not from *B. animalis*), which was able to produce tryptophan, 2-(1H-indol-3-yl)acetate and 2-(1H-indol-3-yl)acetamide and convert 2-(1Hindol-3-yl)acetamide to 2-(1H-indol-3-yl)acetate by an amidase (*Enzyme Commission numbers* [EC] 3.5.1.4). Additionally, more indole metabolite clusters could be generated as depicted in Table 4. Our findings are in line with reports showing that gut microbial species (e.g., *Bacteroides*, *Bifidobacterium*, *Clostridium*, *Peptostreptococcus*, *Lactobacillus*, *Enterobacteria*), produce a variety of tryptophan metabolites such as indole, tryptamine, indoleethanole, indolepropionate, indoleacetate, indolealdeyde, and indoleacriliate [67].

On the other hand, it is well recognized that Indole-derived metabolites have diverse biological activities such as antioxidant properties, antimicrobial effects, modulation of T-cells, and stimulation of enteroendocrine cells through the aryl hydrocarbon receptor (AHR) and pregnane X receptor (PXR) [3,31,32,68,69]. However, information on the effects of indole-derived metabolites on the brain is scarce. In sum, our results suggest that indole metabolites can get into the brain and, due to their high negative docking score, may exert some neuroactive effects via PPARs.

Although PPAR receptors are best-characterized in skeletal muscle, liver, adipose and gut tissue (Figure S5), they can also be found in leukocytes and macrophages as well as in the brain [65,70,71,72,73,74,75,76,77,78,79]. In the CNS, PPARs are expressed in both neurons and glial cells and are widely distributed throughout the brain [80,81,82]. Moreover, their anti-inflammatory effects in the CNS have been documented [83,84,85,86,87].

Based on our results, we hypothesize that *L. rhamnosus* produces tryptophan, 2-(1H-indol-3-yl)acetate and 2-(1H-indol-3-yl)acetamide as key metabolites. As mentioned above, some gut microbial species also produce a variety of tryptophan, 2-(1H-indol-3-yl)acetate and 2-(1H-indol-3-yl)acetamide metabolites as well as 2-(1H-indol-3-yl)acetaldehyde, 2-(1H-indol-3-yl)ethanamine, 2-(1H-indol-3-yl)ethanol, 3-(1H-indol-3-yl)prop-2-enoic acid, 3-(1H-indol-3-yl)prop-2-enoic acid, 3-(1H-indol-3-yl)prop-2-enoic acid, 3-(1H-indol-3-yl)prop-2-enoic acid, 2-(1H-indol-3-yl)-2-oxo-acetic acid, 3-(1H-indol-3-yl)propanoic acid, and 3-(1H-indol-3-yl)-2-oxo-propanoic acid. These metabolites may be absorbed by the GIT and distributed throughout the body, including the brain, and due to their agonist activity at PPARs, may impart anti-inflammatory effects.

Identification of conserved residues in PPARs that are crucial for ligand interaction can provide a detailed map for drug design. For example, previous studies have shown that PPARs interact with a variety of ligands including Kb, fatty acid (FA), and synthetic drugs such as fibrates and thiazolidinediones [62,74,80]. Our findings expand this knowledge by pinpointing specific residues that can be targeted to enhance the efficacy and specificity of PPAR agonists and antagonists (Figure 7 and Figure S3A–E and Table 1, Table 2, Table 3 and Table 4). Thus, our data not only provide some explanation of the mechanism of action of probiotics, but could also suggest novel targets for development of more effective treatments for metabolic disorders such as dyslipidemia, diabetes, obesity, and inflammation.

Our in-silico analysis aimed to decipher at least a hypothetical mechanism of action for bacterial probiotics, and revealed that *L. rhamnosus* metabolites may target PPAR receptors. Therefore, the study of interactions between these indole metabolites and PPARs could guide the design of next-generation PPAR modulators with improved specificity and reduced side effects. Understanding the strong interaction of indole metabolites with PPARs also provides new insights into dietary modulation of PPAR activity, potentially leading to preventive strategies for metabolic diseases and anti-inflammatory effects via diet. Finally, our computational analysis could be expanded to include other species of bacterial probiotics or specific gut microbial composition, as well as their metabolites, producing direct implications for various diseases or protective effects.

In sum, our findings provide compelling evidence that indole metabolites derived from *L. rhamnosus* interact with PPARs, suggesting a potential molecular mechanism by which probiotics exert neuroactive effects such as neuroprotective and/or anti-inflammatory effects. Through in-silico molecular docking analysis, we identified key interactions between these metabolites and conserved residues within PPARs, reinforcing their role in modulating metabolic and immune pathways. These results highlight the relevance of gut microbiota-derived metabolites in regulating nuclear receptor activity, particularly in the context of metabolic and neuroinflammatory disorders.

Furthermore, our study underscores the potential of probiotic-derived metabolites as novel therapeutic agents for modulating PPAR activity, paving the way for the development of targeted dietary and pharmacological interventions. While our computational findings provide valuable insights into the molecular interactions between Indole-derived metabolites and PPARs, further experimental validation through in vitro and in vivo studies is essential to confirm their functional relevance and therapeutic potential. A deeper understanding of these interactions will not only advance our knowledge of the GBA but may allow for better design of next-generation PPAR modulators with enhanced specificity and efficacy.

## 4. Materials and Methods

### 4.1. Database, Webpage and Software

BioCyc Database Collection platform, https://biocyc.org/ (accessed on 1 January 2024); Clustal Omega, https://www.ebi.ac.uk/jdispatcher/msa/clustalo (accessed on 2024, September); DrugBank Online (Academic version), https://go.drugbank.com/ (accessed on 1 September 2024); International Union of Basic and Clinical Pharmacology (IUPHAR)/British Pharmacological Society (BPS) Guide to Pharmacology, https://www.guidetopharmacology.org/ (accessed on 1 September 2024); Jalviwer 2.11.4.0 for Windows, https://www.jalview.org/download/ (accessed on 1 September 2024); KEGG DataBase PPAR signaling pathway. Reference pathway map03320, https://www.genome.jp/pathway/map03320 (accessed on 1 October 2024); KEGG Pathway Maps, https://www.genome.jp/brite/br08901 (accessed on 1 October 2024); Maestro elements Viewer (Version 5.7) by Schrödinger, https://www.schrodinger.com/products/maestro-viewer/ (accessed on 1 August–1 November 2024); Metacyc (https://metacyc.org/) (accessed on 1 January 2024); Protein Data Bank (PDB), https://www.rcsb.org/ (accessed on 1 May 2024); PubChem database platform, https://pubchem.ncbi.nlm.nih.gov/ (accessed on 1 February 2024); PubChem Identifier Exchange Service, https://pubchem.ncbi.nlm.nih.gov/idexchange/idexchange.cgi (accessed on 1 January 2024); Reactome https://reactome.org/ (accessed on 1 April 2024); Schrödinger software https://www.schrodinger.com/ (accessed on 2024, 1 May–1 August 2024, version 2024-1); STRING https://string-db.org (accessed on 1 May 2024); Swiss ADME, http://www.swissadme.ch/ (accessed on 1 March 2024); Swiss Target Prediction (STP) http://www.swisstargetprediction.ch/ (accessed on 1 March 2024); Universal Protein Resource (UniProt), https://www.uniprot.org/ (accessed on 1 May 2024).

### 4.2. Data Collection

With the intention of exploring and proposing a possible mechanism of action of two probiotics, an in-silico study was designed. We identified the metabolites produced by *L. rhamnosus* GG and *B. lactis*, evaluated their ability to cross the BBB, and explored their interactions with target proteins.

#### 4.2.1. Theoretical Metabolites Collection

From the BioCyc DB Collection (https://biocyc.org/ (accessed on 1 January 2024)) [67,88,89,90], we carried out a comparative analysis for the representative genome organism databases, as follows: (Assembly accession numbers: LACTORH) and *B. animalis* (GCF_000025245) (Table S4) and collected metabolites curated from experimentally elucidated metabolic pathways from these probiotic bacteria using Metacyc (https://metacyc.org/ (accessed on 1 January 2024)). On BioCyc, we generated a SmartTable and added SMILES as a property column to accumulate one SMILES code to each metabolite, and then we exported the results to the PubChem Identifier Exchange Service (https://pubchem.ncbi.nlm.nih.gov/idexchange/idexchange.cgi (accessed on 1 January 2024)) to obtain CID and Canonical SMILES codes. SMILES strings contain stereochemical and isotopic specifications, whereas Canonical SMILES strings do not include stereochemical or isotopic information.

#### 4.2.2. Reported Metabolites Collection

We performed an extensive bibliographic search via the PubMed platform using the following keywords: “Metabolomics”, “Analysis”, “*Lactobacillus rhamnosus*”, “*Bifidobacterium animalis*”, “*Bifidobacterium animalis* spp. *lactis* BB12”, “*Bifidobacterium lactis* BB12” and combinations of them to select the metabolites. From the search on PubMed, 22 articles were obtained for the bacterium *L. rhamnosus*, and none for the bacterium *B. animalis* spp. *lactis* BB12. Among the 22 articles, 10 were free access [17,19,25,55,91,92,93,94,95]. Data on metabolites collection was not limited to the growing site of the bacteria or the species in which the bacteria were administered (e.g., rat, mouse, human, or MRD agar or enriched MRD). According to the methodology of the articles, metabolomic analysis of serum, plasma, supernatant of feces or organs or supernatant of bacterial products were performed. Finally, metabolites were manually collected if their number was increased significantly relative to intact controls in the metabolomic analysis.

#### 4.2.3. Natural Metabolites and Reference Drugs of Target Chosen Collection

From DrugBank Online (Academic version) (https://go.drugbank.com/ (accessed on 1 September 2024)) [96] and the International Union of Basic and Clinical Pharmacology (IUPHAR)/British Pharmacological Society (BPS) Guide to Pharmacology (https://www.guidetopharmacology.org/ (accessed on 1 September 2024)) [97], we selected all natural metabolites and reference drugs as docking controls (Table 2 and Table 3). Once we had chosen key targets, we collected natural metabolites and reference drugs.

Data obtained from drug molecules and chemical classification of natural ligands established a robust comparative framework between reference drugs used in clinical settings, natural ligands, and potential compounds. Drugs and natural ligands exhibiting very negative docking values, accompanied by interactions with similar amino acid residues, support the hypothesis that the scores obtained for certain metabolites may be clinically relevant. This reinforces the utility of docking models to predict the biological activity and potential efficacy of new compounds. Furthermore, the identification of metabolites with interactions comparable to those of reference drugs suggests that such compounds could play endogenous modulatory roles in receptor signaling or, alternatively, serve as a basis for the design of new molecules with optimized pharmacological properties.

### 4.3. Target Key Molecules

The number of compounds predicted for each bacterium and the similarity between the mixture of bacteria *L. rhamnosus* and *B. animalis* spp. *lactis* BB12 is depicted in Table 1.

Canonical SMILES strings of all chemical structures of the compounds were collected. Due to the limitations of the analysis platforms, we discriminated compounds with four atoms or fewer, and SMILES strings of above 200 characters [98,99]. In general, 563 SMILES strings were collected from *B. animalis*, 708 from *L. rhamnosus* (1221 metabolites in total) from the BioCyc DataBase, and 266 metabolites from the PubMed review.

#### 4.3.1. Choice of Ligands

Using the Swiss ADME platform (http://www.swissadme.ch/ (accessed on 1 March 2024)), molecules that were not well absorbed in the human GIT or the BBB by passive diffusion were discriminated due to their lipophilicity and polarity, as detailed by the n-octanol/partition co-efficient water (Wlog P) and topological polar surface area (tPSA) [100] (Figure 1, Figure 5 and Figure S4). A total of 688 metabolites collected from the Bio-Cyc model, and 149 from the literature review in PubMed, could be absorbed by the GIT [98,100,101,102] (Figure 5).

In sum, using an in silico computational approach, we identified the metabolites produced by *L. rhamnosus* GG and *B. lactis*, evaluated their ability to cross the BBB, and explored their interactions with target proteins.

#### 4.3.2. Choice of Target Proteins

The canonical SMILES code of each metabolite was put into the Swiss Target Prediction webpage (STP) (http://www.swisstargetprediction.ch/ (accessed on 1 March 2024)) to explore the target proteins of the metabolites. STP predicted the probability of a protein as a target. We obtained 1242 and 867 predicted proteins from the BioCyc and PubMed models, respectively.

#### 4.3.3. Frequency Discrimination

These data were grouped according to cumulative frequency of predicted interaction of the metabolites with the proteins and were ordered from highest to lowest to prepare the cumulative frequency diagrams; the targets that had a frequency above 50% of the maximum frequency were chosen.

Analysis with the STP platform predicted interaction with 62 and 57 target proteins with above 50% of the maximum frequency for the BioCyc and PubMed models, respectively (Figure 2).

#### 4.3.4. Enrichment Analysis Discrimination

With the Universal Protein Resource (UniProt) code, an enrichment analysis of the metabolic pathways was done with the Reactome program (https://reactome.org/ (accessed on 1 September 2024)). A confidence interval of *p* ≤ 0.05 was set as a filter, and disease pathways were included in the analysis to elucidate the cellular, molecular and metabolic pathways in which the target proteins were predicted to participate. A statistical (hypergeometric distribution) test determined whether certain Reactome pathways are over-represented (enriched) in the submitted data. This test produces a probability score, which is corrected for FDR using the Benjamini–Hochberg method. All non-human identifiers have been converted to their human equivalent. IntAct interactors were included to increase the analysis background. This greatly increased the size of Reactome pathways, which maximized the chances of matching the submitted identifiers to the expanded pathways [103].

Using the protein’s common name code, interaction studies were carried out with the STRING program (https://string-db.org (accessed on 1 May 2024)) to create the interaction networks available for *Homo sapiens* and functional enrichment plots. According to the settings, the maps were created following next Network Stats: with minimum interaction scores such as medium (0.4), high (0.7), and very high (0.9), with none or fewer than five interactors to elucidate the protein–protein relationship of the macromolecules identified by the 2D/3D structural prediction. Interactions between proteins are indicated in color-equivalent categories, as follows: experimentally determined (purple), neighboring genes (green), fused genes (red), co-occurrence (dark blue), co-expression (black), database (blue), summary of the scientific literature (text mining, yellow). Supplementary Table S1 shows the parameters for developing the interaction network and Figure 2 illustrates the network obtained with a minimum interaction score of 0.7 [104].

### 4.4. Docking Analysis

Docking analysis utilized Schrödinger software (https://www.schrodinger.com/ (accessed on 1 May–1 August 2024)) and a detailed analysis performed with Maestro elements Viewer (Version 5.7) by Schrödinger (https://www.schrodinger.com/products/maestro-viewer/ (accessed on 1 August–1 November 2024)) (Schrödinger Release 2024-1: Maestro, Schrödinger, LLC, New York, NY, USA, 2025).

#### 4.4.1. Metabolites, Ligand, and Protein Preparation

Metabolites and ligands were optimized under physiological conditions at pH 7.4 in LigPrep [Schrödinger, LLC (2021). Schrödinger Release 2025-1: LigPrep, Schrödinger, LLC, New York, NY, USA, 2025]. The proteins were prepared under physiological conditions in Protein Preparation Wizard Schrödinger Release 2025-1: Protein Preparation Workflow [105]; Epik, Schrödinger, LLC, New York, NY, USA, 2024; Impact, Schrödinger, LLC, New York, NY, USA; Prime, Schrödinger, LLC, New York, NY, USA, 2025.

#### 4.4.2. Ligand Structure Obtention

The 2D conformers in SDF format of all metabolites were obtained. Canonical SMILES, STP and SADME were used in analysis of CID on the PubChem Identifier Exchange Service (https://pubchem.ncbi.nlm.nih.gov/idexchange/idexchange.cgi (accessed on 1 January 2024)), and on the PubChem database platform [106].

#### 4.4.3. Protein Structure Obtention

Protein crystal codes were selected from Protein Data Bank (PDB); https://www.rcsb.org/ (accessed on 1 May 2024)). The following protein crystal codes were used for the peroxisome proliferator-activated receptor (PPAR) and the retinoic acid receptor (RXR) protein. PPARA: PDB 3G8I and resolution 2.2Å; PPAR B/D: PDB: 7VWF, 1.9 Å; PPARG: PDB: 8B92, 1.66 Å; RXRA: PDB: 7A77, 1.5 Å; RXRB: PDB: 7A78, 1.72 Å; RXRG: PDB: 7A79, 2.5 Å. The interactions were classified by color according to their nature, as follows: gray: any interaction; gold: with skeleton; turquoise: with side chain; green: with hydrophobic residues (PHE, LEU, ILE, TYR, TRP, VAL, MET, PRO, CYS, ALA, CYX); purple: with residues that have aromatic rings (PHE, TYR, TRP, TYO); orange: with polar residues (ARG, ASP, GLU, HIS, ASN, GLN, LYS, SER, THR, ARN, ASH, GLH, HID, HIE, LYN); magenta: with charged polar residues (ARG, ASP, GLU, LYS, HIP, CYT, SRO, TYO, THO); blue: with hydrogen bond donors; red: with hydrogen post acceptors; white: no interaction [52,102,107,108].

### 4.5. Statistical Analysis, Validation and Exclusion Criteria

Statistical analyses were performed using a combination of parametric and non-parametric methods to account for data distribution and sample size. Group differences in docking scores were assessed via two-way ANOVA followed by Tukey’s post-hoc test (parametric) and Kruskal–Wallis test with Dunn’s correction (non-parametric). Boxplots depict median docking scores (central line), 5th–95th percentiles (whiskers), and interquartile ranges (boxes), with significance levels set at * *p* < 0.05, ** *p* < 0.01, and *** *p* < 0.001. Non-normal residuals were observed, but the robust sample size supported the validity of parametric inferences. GraphPad Prism 10 has been used to analysis.

Analyte exclusion criteria included: (1) docking scores below the 5th percentile of cumulative frequency distributions; (2) metabolites non-distributed to brain; and (3) pathways with FDR-adjusted *p* > 0.05. Disease pathways were prioritized to elucidate molecular and metabolic mechanisms linked to target proteins.

Target inclusion criteria included targets exceeding 50% of the maximum.

Target exclusion criteria included: (1) targets with less than 50% of the maximum, (2) lack of interactions, (3) carbonic anhydrase due to its minimal interaction with nuclear receptors.

Protein–protein interaction networks and functional enrichment were analyzed using STRING for *Homo sapiens*, with interaction scores filtered at medium (0.4), high (0.7), and very high (0.9) confidence thresholds. Pathways were validated via overrepresentation analysis in Reactome, employing a hypergeometric test to identify enriched pathways. The Benjamini–Hochberg method was used to adjust for FDR. Non-human identifiers were mapped to human orthologs, and IntAct interactors were included to expand the background dataset.

## 5. Conclusions

The main findings implicate PPARs/RXRs in the neuroactive effects of specific probiotics. Based on the biological pathways and cellular expression of PPARs, indole metabolites may play a role in metabolic regulation, cell differentiation, survival, and inflammatory responses. Furthermore, we identified key conserved residues in PPARs that are essential for ligand interactions, which could facilitate the development of PPAR-targeted drugs or probiotic-based therapies for various neurological diseases.

## Figures and Tables

**Table 1:** Heat map of Docking score of metabolites with the capacity to cross the BBB with a ranking above the 95th percentile in binding affinity to nuclear receptors (−8.12 for PPARA, −7.13 for PPARB, −7.17 for PPARG, −7.28 for RXRA, −6.94 for RXRB and −7.00 for RXRG). Scale indicates docking score according to a specific color: green: −10 (strong docking score), yellow: −5 (medium docking score) and red: zero (no docking).

BioCyc								
**CID**	**Trivial Name**	**PPARA**	**PPARB**	**PPARG**	**RXRA**	**RXRB**	**RXRG**	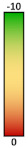
	Limit of 95% percentile	−8.13	−7.05	−7.52	−7.14	−6.85	−6.84	
397	Indole-3-acetamide	−8.22	−7.61		−7.97	−7.98	−7.83	
995	Phenanthrene		−7.31		−8.54	−8.50	−8.48	
6780	Anthraquinone		−7.48		−8.02	−8.23	−8.09	
6986	P-menthan-3-one					−7.00	−6.80	
7108	Phenothiazine		−7.12		−8.11	−8.09	−7.91	
7460	Alpha-phellandrene						−7.05	
8400	Benzoin		−7.05		−7.80	−7.36	−7.22	
11142	Beta-phellandrene						−6.80	
26049	3-carene					−7.01	−7.10	
28649	Stilbene oxide		−7.10		−7.13		−7.25	
62349	Menthone lactone		−7.16		−7.25	−7.24	−7.18	
70117	3-chlorobenzyl alcohol					−6.94		
234817	Pinoresinol			−8.17				
363863	Maackiain			−7.90	−7.84			
439901	4′-o-methylisoflavone	−8.25	−7.33	−7.73			−7.10	
445354	Retinol; vitamin a			−9.02	−7.99	−8.18	−8.13	
494912	Nsc636229			−7.55		−7.55	−7.01	
623060	Medicarpin(p)			−8.06	−7.06			
3326923	Ibuprofen anion	−8.71	−7.76					
4055279	1-phenylpropan-2-ylazanium					−6.85		
5377291	Hinokiresinol		−7.29		−7.32	−6.96	−7.33	
6440617	(z)-hinokiresinol			−8.52	−8.78	−7.71	−7.06	
10966551	4′-hydroxyisoflavone		−7.63	−8.05	−7.81	−7.33	−7.86	
25246088	Cis-12,13-epoxy-9-octadecenoic acid				−7.30		−7.23	
77916059	Noroxomaritidine				−8.48	−8.62	−6.92	
146037227	Oxomaritinamine				−7.99	−7.81		
PubMed								
CID	Trivial Name	PPARA	PPARB	PPARG	RXRA	RXRB	RXRG	
	Limit of 95% percentile	−8.12	−7.13	−7.17	−7.28	−6.94	−7.00	
322	4-trans-4-Hydroxycinnamic acid				−7.29			
800	2-(1H-indol-3-yl)acetaldehyde		−6.99		−7.23	−7.23	−7.16	
803	Indole-3-pyruvic acid	−8.79	−7.82	−7.24		−7.05	−7.01	
1150	Tryptamine		−8.01		−7.50	−7.66	−7.23	
3744	3-Indolepropionic acid	−8.60	−7.50			−6.85		
10394	3-(4-Hydroxyphenyl)propionic acid				−7.40			
10685	Tryptophol					−6.98	−7.10	
14558	Indol-3-acrylic acid	−8.26	−7.07			−6.89		
73863	3-Indoleglyoxylic acid	−8.02	−7.40	−7.10		−6.92		

**Table 2:** Heat map of Docking score of natural metabolites and Indole metabolites. Scale indicates docking score according to a specific color; green: −10 (strong docking score), yellow: −5 (medium docking score) and red: zero (no docking). BBB: blood–brain barrier; DHA: Docosahexaenoic acid; EPA: Eicosapentaenoic acid; Kb: Ketone bodies; LCFA: long chain fatty acid; MCFA: medium chain fatty acid; MUFA: mono-unsaturated fatty acid; PUFA: poly-unsaturated fatty acid; SCFA: short chain fatty acid.

Vit. A	CID	Name	PPARA	PPARB	PPARG	RXRA	RXRB	RXRG	
1	445354-BBB	Retinol	−7.014	−7.825	−9.024	−7.992	−8.181	−8.129	** 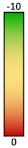 **
2	6419707	Retinoate	−8.453	−7.825	−7.543	−8.310	−9.383	−9.680	
3	638015	Retinal	−8.021	−6.080	−7.497	−8.913	−7.593	−7.880	
4	449171-BBB	Retinoic acid				−9.790	−9.700	−9.509	
Kb	CID	Name	PPARA	PPARB	PPARG	RXRA	RXRB	RXRG	
1	4071895	2-hydroxybutyrate	−6.327	−5.505	−5.391	−5.829	−5.428	−5.542	
2	180	acetone	−5.023	−4.554	−4.615	−4.373	−4.389	−4.490	
3	6971017	acetoacetate	−6.571	−5.395	−6.229	−6.382	−5.711	−5.823	
SCFA	CID	Name	PPARA	PPARB	PPARG	RXRA	RXRB	RXRG	
1	283	Methanoate (Formiate)	−5.019	−3.795	−4.432	−3.392	−3.427	−4.199	
2	175	Ethanoate (Acetate)	−5.573	−4.312	−4.934	−5.303	−4.643	−4.762	
3	176	Ethanoic (Acetic) acid	−5.573	−4.312	−4.934	−5.303	−4.643	−4.762	
4	104745	Propanoate	−4.115	−2.710	−3.432	−4.449	−3.204	−4.131	
5	1032-BBB	Propanoic (Propionic) acid	−4.112	−2.708	−3.430	−4.447	−3.202	−4.129	
6	264-BBB	Butanoic (Butyric) acid	−6.320	−5.000	−5.590	−6.094	−5.576	−5.670	
7	7991-BBB	Pentanoic (Valeric) acid	−5.633	−4.942	−4.846	−5.577	−4.631	−5.271	
8	8892-BBB	Hexanoic acid (Caproic acid)	−5.126	−3.836	−4.998	−5.347	−4.956	−4.486	
MCFA	CID	Name	PPARA	PPARB	PPARG	RXRA	RXRB	RXRG	
1	119389-BBB	Octanoate (Caprylate)	−5.318	−3.839	−4.974	−5.415	−4.839	−5.405	
2	379-BBB	Octanoic (caprylic) acid	−5.315	−3.836	−4.971	−5.412	−4.836	−5.402	
3	8158-BBB	Nonanoic (pelargonic) acid	−5.559	−4.129	−3.245	−5.725	−4.448	−5.551	
4	2969-BBB	Decanoic (capric) acid	−2.473	−1.548	−3.480	−3.678	−3.478	−3.765	
5	4149208-BBB	Dodecanoate (laurate)	−2.521	−2.652	−1.423	−4.770	−3.904	−4.077	
LCFA	CID	Name	PPARA	PPARB	PPARG	RXRA	RXRB	RXRG	
1	11005-BBB	Tetradecanoic (Myristic) acid	−2.847	−1.169	−1.731	−4.993	−4.607	−4.769	
2	13849-BBB	pentadecanoic acid	−2.991	−1.823	−1.844	−5.786	−5.031	−5.088	
3	504166-BBB	Hexadecanoate (Palmitate)	−3.578	−2.276	−1.883	−5.878	−5.398	−5.662	
4	985-BBB	Hexadecanoic (Palmitic acid)	−3.575	−2.273	−1.880	−5.875	−5.395	−5.659	
5	10465-BBB	Heptadecanoic acid	−4.241	−2.703	−2.175	−6.517	−6.103	−6.162	
6	3033836	Octadecanoate (Stearate)	−3.581	−3.511	−2.928	−3.219	−1.485	−2.856	
7	5281	Octadecanoic (Stearic) acid	−3.578	−3.508	−2.925	−3.216	−1.482	−2.853	
8	12591	Nonadecanoic acid	−3.937	−1.175	−2.888	−3.040	−1.611	−2.890	
MUFA	CID	Name	PPARA	PPARB	PPARG	RXRA	RXRB	RXRG	
1	19499-BBB	but-2-enoic acid	−4.408	−3.736	−4.482	−4.874	−4.560	−4.792	
2	19499-BBB	but-2-enoic acid	−4.126	−3.308	−3.926	−4.709	−3.794	−3.850	
3	151007-BBB	dodec-5-enoic acid	−5.090	−2.392	−4.630	−4.986	−4.335	−4.942	
4	151007-BBB	(Z)-dodec-5-enoic acid (Lauroleinic acid)	−3.088	−2.366	−1.959	−4.607	−4.275	−4.506	
5	5461012-BBB	(Z)-hexadec-9-enoate (Palmitoleate)	−3.621	−2.500	−2.775	−5.762	−5.211	−6.290	
6	4668-BBB	hexadec-9-enoic acid	−4.056	−2.681	−2.772	−6.277	−5.741	−5.630	
7	4668-BBB	hexadec-9-enoic acid (Palmitoleic acid)	−3.618	−2.497	−2.538	−5.759	−5.208	−6.287	
8	5461069	(Z)-octadec-11-enoate (Vaccenate)	−4.755	−2.254	−3.408	−6.545	−6.377	−6.270	
9	5460221-BBB	(Z)-octadec-9-enoate (Oleate)	−4.130	−3.831	−2.946	−3.632	−3.445	−6.620	
10	12745	octadec-10-enoic acid	−4.334	−2.947	−3.766	−7.002	−5.919	−5.977	
11	12745	octadec-10-enoic acid (cis-10-Oleic acid)	−3.945	−2.531	−3.234	−6.013	−5.900	−4.571	
12	965	octadec-9-enoic acid	−4.777	−3.983	−2.943	−6.762	−3.442	−6.617	
13	965	octadec-9-enoic acid (cis-9-Oleic acid)	−4.127	−3.828	−3.848	−3.629	−6.084	−6.617	
PUFA	CID	Name	PPARA	PPARB	PPARG	RXRA	RXRB	RXRG	
1	3931-BBB	(9Z,12Z)-octadeca-9,12-dienoic acid (Linoleic acid)	−5.805	−3.795	−6.315	−7.147	−6.515	−6.805	
2	3931-BBB	octadeca-9,12-dienoic acid	−5.435	−3.479	−5.907	−7.089	−6.513	−6.43	
3	3931-BBB	octadeca-9,12-dienoic acid	−5.342	−2.787	−5.761	−7.02	−6.476	−6.394	
4	3931-BBB	octadeca-9,12-dienoic acid	−5.141	−2.386	−3.474	−6.558	−6.263	−6.364	
5	860-BBB	octadeca-9,12,15-trienoic acid	−6.388	−4.839	−6.407	−7.015	−6.713	−6.859	
6	860-BBB	octadeca-9,12,15-trienoic acid	−5.267	−4.74	−6.128	−7.001	−6.539	−6.804	
7	860-BBB	octadeca-9,12,15-trienoic acid	−5.123	−4.262	−3.892	−6.772	−6.51	−6.712	
8	860-BBB	octadeca-9,12,15-trienoic acid	−4.992	−4.247	−3.867	−6.74	−6.301	−6.548	
9	860-BBB	octadeca-9,12,15-trienoic acid	−4.992	−4.16	−3.307	−6.62	−5.864	−6.526	
10	860-BBB	(9Z,12Z,15Z)-octadeca-9,12,15-trienoic acid (alpha-Linolenic acid)	−4.973	−4.129	−3.286	−6.501	−5.773	−6.265	
11	860-BBB	octadeca-9,12,15-trienoic acid	−4.786	−2.834	−3.028	−6.173	−5.633	−6.028	
12	860-BBB	octadeca-9,12,15-trienoic acid	−4.58	−2.706	−3.001	−3.615	−4.045	−5.125	
	445580	(4Z,7Z,10Z,13Z,16Z,19Z)-docosa-4,7,10,13,16,19-hexaenoic acid (DHA)	−7.014	−7.416	−8.724	−8.746	−9.264	−9.512	
	446284	(5Z,8Z,11Z,14Z,17Z)-eicosa-5,8,11,14,17-pentaenoic acid (EPA)	−8.815	−7.433	−6.911	−8.441	−6.812	−7.845	
			PubMed						
Indole	CID	Name	PPARA	PPARB	PPARG	RXRA	RXRB	RXRG	
1	798-BBB	indole	−5.934	−5.692	−5.840	−6.131	−6.164	−6.177	
2	10256-BBB	1H-indole-3-carbaldehyde	−7.272	−6.528	−6.521	−6.647	−6.598	−6.685	
3	800-BBB	2-(1H-indol-3-yl)acetaldehyde	−7.715	−6.991	−7.035	−7.227	−7.234	−7.160	
4	1150-BBB	2-(1H-indol-3-yl)ethanamine	−7.231	−8.007	−6.737	−7.497	***−7.657***	−7.232	
5	10685-BBB	2-(1H-indol-3-yl)ethanol	−6.942	−6.926	−6.405	−7.085	−6.977	−7.102	
6	14558-BBB	3-(1H-indol-3-yl)prop-2-enoic acid	−8.258	−7.067	−6.570	−6.679	−6.889	−6.594	
7	14558-BBB	3-(1H-indol-3-yl)prop-2-enoic acid	−7.733	−6.943	−6.094	−6.329	−6.251	−5.938	
8	14558-BBB	3-(1H-indol-3-yl)prop-2-enoic acid	−5.135	−4.448	−4.461	−5.225	−5.464	−5.033	
9	14558-BBB	3-(1H-indol-3-yl)prop-2-enoic acid	−4.749	−3.815	−3.898	−4.963	−4.891	−4.967	
10	73863-BBB	2-(1H-indol-3-yl)-2-oxo-acetic acid	−8.016	−7.400	−7.097	−7.119	−6.917	−6.937	
11	3744-BBB	3-(1H-indol-3-yl)propanoic acid	−8.599	−7.499	−6.597	−6.801	−6.851	−6.737	
12	803-BBB	3-(1H-indol-3-yl)-2-oxo-propanoic acid	−8.790	−7.815	−7.243	−7.006	−7.045	−7.008	
13	6932058	1H-indole-3-carboxylate	−7.624	−6.243	−6.505	−6.643	−6.749	−6.465	
14	801	2-(1H-indol-3-yl)acetate (Indolacetate)	−7.827	−7.306	−6.719	−7.047	−7.065	−7.283	
15	3080590	2-(2-oxoindolin-3-yl)acetic acid	−7.619	−6.742	−7.175	−7.063	−7.151	−6.989	
16	92904	2-hydroxy-3-(1H-indol-3-yl)propanoic acid	−8.578	−8.014	−6.787	−7.225	−7.066	−7.210	

DHA: Docosahexaenoic acid; EPA: Eicosapentaenoic acid; Kb: Ketone bodies; LCFA: long chain fatty acid; MCFA: medium chain fatty acid; MUFA: mono-unsaturated fatty acid; PUFA: poly-unsaturated fatty acid; SCFA: short chain fatty acid. Heat map indicates docking score. More negative (green) interactions are stronger. Red interactions (near to zero) are weaker.

**Table 3:** Docking score of drug reference molecules. Scale indicates docking score according to a specific color; green: 10 (strong docking score), yellow: −5 (medium docking score) and red: zero (no docking).

Action	CID	Trivial Name	GIT	BBB	IUPHAR/BPS	DB	PPARA	PPARB	PPARG	RXRA	RXRB	RXRG	
Antagonist (Anta)	446738	GW 6471	Low	No	Yes	No	−8.79						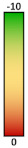
							−8.51						
							−8.35						
	46233311	GSK0660	Low	No	Yes	No		−5.21					
								−4.87					
	82146	Bexarotene	High	No	Yes	Yes			−9.16				
	445154	Resveratrol	High	Yes	Yes	Yes			−8.46				
									−4.53				
	3033	Diclofenac	High	Yes	Yes	Yes			−7.75				
Agonist (Ago)	3339	Fenofibrate	High	Yes	Yes	Yes	−7.82						
	2796	Clofibrate	High	Yes	Yes	Yes	−6.50						
	9864881	Elafibranor	High	No	Yes	Yes	−6.57						
	9891946	L-796449	Low	No	Yes	No		−9.24					
	11236126	Seladelpar	Low	No	Yes	Yes		−8.15					
	4075	Mesalamine	High	No	Yes	Yes			−5.83				
	3715	Indomethacin	High	Yes	Yes	Yes			−9.25				
	4829	Pioglitazone	High	No	Yes	Yes			−8.19				
									−7.71				
									−6.71				
									−5.90				
	77999	Rosiglitazone	High	No	Yes	Yes			−8.13				
									−7.76				
									−7.37				
									−7.01				
	9864881	Elafibranor	High	No	Yes	Yes			−7.29				
Antagonist (Anta)	1548887	Sulindac	High	No	Yes	Yes				−8.88			
	3922	LG-100268	High	Yes	Yes	No				−9.43			
	3922	LG-100268	High	Yes	Yes	No					−8.85		
	3922	LG-100268	High	Yes	Yes	No						−8.26	
Agonist (Ago)	25195496	Fluorobexarotene	High	No	Yes	No				−10.60			
Activator	82146	Bexarotene	High	No	Yes	Yes				−9.92			
	82146	Bexarotene	High	No	Yes	Yes					−10.06		
	82146	Bexarotene	High	No	Yes	Yes						−10.22	

**Table 4:** Sum of specific aminoacidic residues on agonist or antagonist control of PPARs and RXRs. Blood: residue that interact >75% residues; normal: residue that interact >75% residues; green: residues that dock with agonist; red: residues that dock with antagonist; yellow; residues that only appear in one protein isotype.

***A. Alignment numbering***		*26*	*29*	*59*	62	63	65	66	*67*	*78*	**85**	**86**	87	88	**89**	91	95	*96*	**123**	126	127	130	139	141	*152*	*156*	*160*	**163**	164	167	**249**	253	*265*	**292**	**273**
***Residues dock agonist***	***PPARA***	-	-	-	-	-	-	-	-	-	**C276**	**Q277**	-	-	**S280**	-	-	-	**Y314**	-	F318	-	M330	V332	-	-	-	**I354**	K358	-	**H440**	V444	-	**L460**	**Y464**
	***PPARB***	-	-	-	-	-	-	-	-	-	**C285**	Q286	-	-	**T289**	-	-	-	H323	-	F327	-	L330	V341	-	-	-	I363	I364	-	**H449**	M453	-	L469	Y473
	***PPARG***	-	-	-	-	-	-	-	-	-	**C285**	-	-	**R288**	S289	-	-	-	-	L326	Y327	L330	-	L341	-	-	-	-	-	-	-	-	-	-	-
***Residues dock antagonist***	***PPARA***	-	-	-	T253	L254	A256	K257	-	-	-	-	C278	-	-	E282	-	-	-	-	-	-	-	-	-	-	-	-	-	-	-	-	-	-	-
	***PPARB***	-	-	-	-	-	-	-	-	-	-	-	C287	-	-	E291	E295	-	-	-	-	-	-	-	-	-	-	-	-	-	-	-	-	-	-
	***PPARG***	-	-	-	-	-	-	-	-	-	-	-	-	-	-	-	-	-	-	-	-	-	-	-	-	-	-	-	-	-	-	-	-	-	-
***Specific residues***	***PPARA***	-	M220	E251	T253	L254	A256	-	L258	-	-	-	-	-	-	-	-	-	-	-	-	-	-	-	-	-	-	-	-	-	-	-	L456	-	-
	***PPARB***	-	-	-	-	-	-	-	-	-	-	-	-	-	-	-	-	-	-	-	-	-	-	-	F352	-	-	-	-	-	-	-	-	-	-
	***PPARG***	F226	-	-	-	-	-	-	-	A278	-	-	-	-	-	-	-	I296	-	-	-	-	-	-	-	L356	F360	-	-	-	-	-	-	-	-
Blood: residue that interact >75% residues; green: residues that dock with antagonist; Red: residues that dock with agonist; yellow: specific PPAR residues that dock with any molecule.																																			
***B. Alignment numbering***		50	54	88	91	92	95	124	127	128	131	214	217	218	221																				
***Agonist***	***RXRA***	**I268**	A272	N306	L309	-	**F313**	V342	**L345**	F346	V349	**C432**	H435	**L436**	**F439**																				
	***RXRB***	**L339**	A343	**N377**	-	I381	**F384**	V413	**I416**	F417	V420	**C503**	H506	**L507**	F510																				
	***RXRG***	**V266**	I269	**N307**	-	L311	**F314**	V343	**L346**	F347	V350	**C433**	H436	**L437**	**F440**																				
Blood: residue that interact >75% residues; normal: residue that interact >75% residues; green: residues that dock with agonist; red: residues that dock with antagonist; yellow; residues that only appear in one protein isotype.																																			

## Data Availability

The raw data supporting the conclusions of this article will be made available by the authors, without undue reservation.

## Supplementary Materials

The following supporting information can be downloaded at: https://www.mdpi.com/article/10.3390/ijms26104507/s1.

## Nomenclature

AHR	Aryl Hydrocarbon Receptor
ADME	Absorption, Distribution, Metabolism, and Excretion
AF-2 helix	Activation function-2 helix
Ago	Agonist
AKT	Protein kinase B
Ant	Antagonist
*B. animalis*	*Bifidobacterium animalis* spp. *lactis*
*B. lactis*	*Bifidobacterium animalis* spp. *lactis*
BBB	Blood-Brain Barrier
BioCyc DB	BioCyc Database Collection
CA	Carbonic anhydrase
CFU	Colony-forming units
CID	Compound Identifier (PubChem)
CNS	Central Nervous System
DHA	Docosahexaenoic acid, a polyunsaturated fatty acid (PUFA)
EC	EC Number
EPA	Eicosapentaenoic acid, a polyunsaturated fatty acid (PUFA)
FA	Fatty Acids
FDR	False discovery rate
FMT	Fecal microbiota transplantation
GABA	Gamma-aminobutyric acid
GBA	Gut-Brain Axis
GIT	Gastrointestinal Tract
GM	Gut Microbiota
HGNC	Human Gene Nomenclature Committee
HIE	Polar histidine
KB	Ketone Bodies
LCFAs	Long-Chain Fatty Acids
LBD	Ligand-binding domain
*L. rhamnosus*	*Lacticaseibacillus rhamnosus*
MAPK	Mitogen-activated protein kinases
MCFA	Medium-chain fatty acid
MUFAs	Monounsaturated Fatty Acids
NCOA1	Nuclear Receptor Coactivator 1
NOTCH	Eurogenic locus notch homolog protein
PD	Parkinson Disease
PubMed DB	PubMed Database
PXR	Pregnane X Receptor
PPARs	Peroxisome Proliferator-Activated Receptors
PPARA	Peroxisome Proliferator-Activated Receptor Alpha
PPARB/D	Peroxisome Proliferator-Activated Receptor Beta/Delta
PPARG	Peroxisome Proliferator-Activated Receptor Gamma
PUFAs	Polyunsaturated Fatty Acids
RXRA	Retinoid X Receptor Alpha
RXRB	Retinoid X Receptor Beta
RXRG	Retinoid X Receptor Gamma
SDF	Structure Data File
SMILES	Simplified Molecular Input Line Entry Specification
SFA	Saturated Fatty Acids
SCFAs	Short-Chain Fatty Acids
STP	Swiss Target Prediction
STRING	Search Tool for the Retrieval of Interacting Genes/Proteins
TGF	Transforming growth factor beta receptor
TMAO	Tryptophan metabolites, trimethylamine-N-oxide
tPSA	Topological polar surface area
TRPV	Transient receptor potential cation channel subfamily V member
UNIPROT	Universal Protein Resource
Wlog P	n-octanol/partition co-efficient water
