# Supplementary material for: Peroxisome Proliferator-Activated Receptors (PPARs) May Mediate the Neuroactive Effects of Probiotic Metabolites: An In Silico Approach
Source: Int J Mol Sci. 2025 May 9;26(10):4507. doi: 10.3390/ijms26104507 (PMC12111801; doi:10.3390/ijms26104507)
Supplement: Supplementary file 1 [file ijms-26-04507-s001.zip › 02 Supplementary material. Figures full quality.pdf]

Article

Peroxisome proliferator-activated receptors (PPARs) may mediate the neuroactivity effects of probiotic metabolites. An in-silico approach

Irving Parra[1], Alan Carrasco-Carballo[2], Victoria Palafox[3], Isabel Martínez-García[1], José Aguilera[4], José Luis Góngora Alfaro[5], Irma Isela Aranda-González[6], Yousef Tizabi[7], Liliana Mendieta1*

[1]Laboratorio de Neuroquímica, Facultad de Ciencias Químicas Benemérita Universidad Autónoma de Puebla, Puebla, 72570, México
[2]Laboratorio de Elucidación y Síntesis en Química Orgánica, Facultad de Ciencias Químicas, Benemérita Universidad Autónoma de Puebla, Puebla, México
[3]Institute for Obesity Research, Instituto Tecnológico y de Estudios Superiores de Monterrey, Monterrey, México
[4]Inst. de Neurociències, Univ. Autònoma de Barcelona, Cerdanyola del Vallès, Spain
[5]Departamento de Neurociencias, Centro de Investigaciones Regionales ''Dr. Hideyo Noguchi", Universidad Autónoma de Yucatán, Avenida Itzáes No. 490 x 59, Mérida, Yucatán 97000, México
[6]Facultad de Medicina, Universidad Autónoma de Yucatán, Avenida Itzáes No. 498 x 59 y 59A, Mérida, Yucatán 97000, México
[7]Department of Pharmacology, Howard University College of Medicine, Washington, DC. USA

*Correspondence: liliana.martinezmen@correo.buap.mx

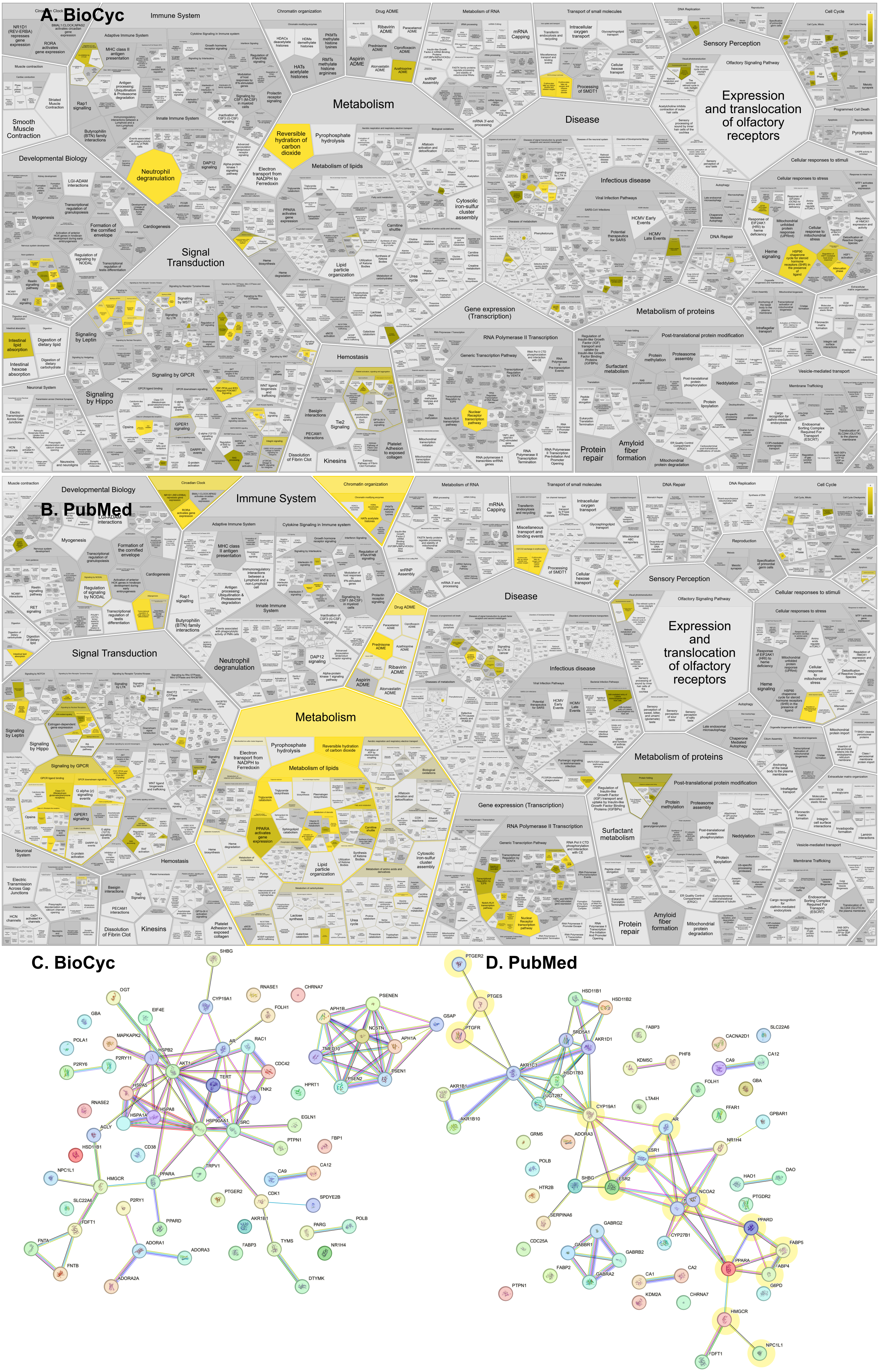
A. BioCyc
Immune System
Metabolism
Disease
Expression and translocation of olfactory receptors
Developmental Biology
Signal Transduction
Neutrophil degranulation
Reversible hydration of carbon dioxide
Metabolism of lipids
Infectious disease
Metabolism of proteins
Gene expression (Transcription)
Hemostasis
Signaling by GPCR
Sensory Perception
Cell Cycle
Cellular responses to stimuli
B. PubMed
Immune System
Developmental Biology
Signal Transduction
Neutrophil degranulation
Metabolism
Metabolism of lipids
Disease
Expression and translocation of olfactory receptors
Infectious disease
Metabolism of proteins
Gene expression (Transcription)
Hemostasis
Sensory Perception
Cell Cycle
Cellular responses to stimuli
C. BioCyc
SHBG
OGT
GBA
CYP19A1
RNASE1
CHRNA7
FOLH1
PSENEN
APH1B
GSAP
NCSTN
APH1A
TMED10
PSEN2
PSEN1
EIF4E
MAPKAPK2
POLA1
P2RY6
P2RY11
HSPB2
AKT1
AR
RAC1
CDC42
TERT
TNK2
HPRT1
HSPA5
HSPA1A
HSPA8
RNASE2
HSP90AA1
SRC
EGLN1
ACLY
PTPN1
FBP1
HSD11B1
CD38
TRPV1
CA12
NPC1L1
HMGCR
PPARA
CA9
SLC22A6
P2RY1
PTGER2
CDK1
SPDYE2B
FDFT1
PPARD
AKR1B1
POLB
PARG
FNTA
ADORA1
ADORA3
FABP3
TYMS
NR1H4
FNTB
ADORA2A
DTYMK
D. PubMed
PTGER2
PTGES
PTGFR
HSD11B1
HSD11B2
SRD5A1
AKR1D1
AKR1C3
HSD17B3
AKR1B1
UGT2B7
AKR1B10
FABP3
CACNA2D1
SLC22A6
KDM5C
PHF8
CA9
CA12
FOLH1
GBA
LTA4H
FFAR1
CYP19A1
AR
GPBAR1
GRM5
ADORA3
NR1H4
ESR1
POLB
SHBG
ESR2
HTR2B
HAO1
DAO
NCOA2
PTGDR2
SERPINA6
PPARD
GABRG2
CYP27B1
FABP5
CDC25A
GABRB1
GABRB2
PPARA
FABP4
FABP2
GABRA2
G6PD
CA1
CA2
PTPN1
KDM2A
CHRNA7
HMGCR
NPC1L1
FDFT1

Figure S1. Voronoi diagrams to Reactome and Interactome from BioCyc and PubMed. Voronoi diagrams of the cellular, molecular and metabolic pathways are shown in which the predicted target proteins for the metabolites produced by the bacteria *L. rhamnosus* LGG and *B. animalis* spp *lactis* BB12 (BioCyc; A) metabolites derived from the bibliographic review (PubMed; C) metabolites with a confidence interval less than or equal to 0.05. C and D shows interaction networks of the predicted target proteins for the metabolites produced by the bacteria *L. rhamnosus* LGG and *B. animalis* spp *lactis* BB12 according to the BioCyc database (A) and the metabolites derived from the literature review in PubMed and Cumulative frequency graphs discrimination. Metabolites with a minimum interaction score of 0.7 and maximum of 5 interactors. Validation model and Network Stats are shown at Supplementary Table 1. Interactions between proteins are indicated in color-equivalent categories lines, namely: experimentally determined (purple), neighboring genes (green), fused genes (red), co-occurrence (dark blue), co-expression (black), database (blue clear), summary of the scientific literature (text mining, yellow). Yellow circles: Protein implicates in metabolic pathways.

Table S1. Validation model and Network Stats

Model	***BioCyc***					
Confidence interval	***0.4 (Medium)***		***0.7 (High)***		***0.9 (Highest)***	
Interactors	None	Max 5	None	Max 5	None	Max 5
Number of nodes	60	65	60	65	60	65
Number of links	90	236	65	89	39	55
Number of links expected	86	102	20	28	8	14
PPI enrichment value	$p < 1{,}0 \times 10^{-16}$	$p < 1{,}0 \times 10^{-16}$	$p < 3.89 \times 10^{-15}$	$p < 1{,}0 \times 10^{-16}$	$p < 5.55 \times 10^{-15}$	$p < 1{,}0 \times 10^{-16}$
Model	**PubMed**					
Confidence interval	***0.4 (Medium)***		***0.7 (High)***		***0.9 (Highest)***	
Interactors	None	Max 5	None	Max 5	None	Max 5
Number of nodes	47	82	47	52	47	52
Number of links	106	138	40	62	12	24
Number of links expected	17	30	3	10	1	7
PPI enrichment value	$p < 1{,}0 \times 10^{-16}$	$p < 1{,}0 \times 10^{-16}$	$p < 1{,}0 \times 10^{-16}$	$p < 1{,}0 \times 10^{-16}$	$p < 7.9 \times 10^{-10}$	$p < 1.59 \times 10^{-7}$

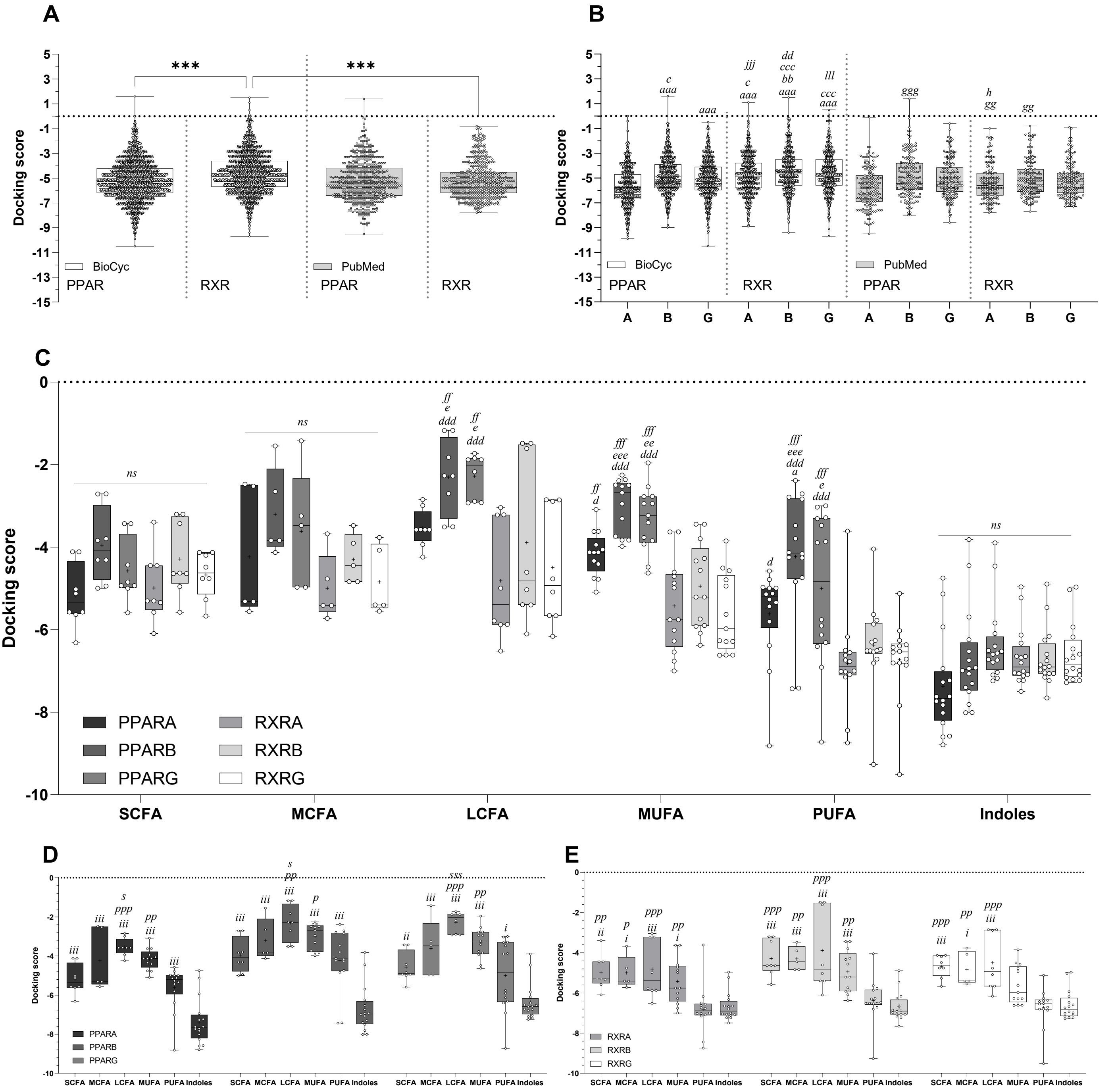

Figure S2. Comparative analysis of ligand-receptor docking score distributions across PPAR and RXR isoforms. (A) Dataset comparisons (BioCyc vs. PubMed) reveal stronger ligand interactions for PPAR-BioCyc compared to RXR-BioCyc (*p < 0.001*), while RXR-PubMed exhibits enhanced binding over RXR-BioCyc (*p < 0.001*) Kruskal-Wallis followed by Dunn's test was performance to detect differences among groups and significance level are ****p < 0.001*. (B) Isoform-specific analysis identifies PPARA-BioCyc as the highest-affinity receptor across all PPAR and RXR isoforms (*p < 0.001*), with PPARG-BioCyc outperforming PPARB-BioCyc and all RXR isoforms. PPARA-PubMed shows superior binding to PPARB-PubMed and RXRA/B-PubMed (*p < 0.01*). Two-way ANOVA followed by Tukey test was performance to detect differences among groups. Boxplots depict median docking scores, 5th–95th percentiles, and significance level are (**p < 0.05*, ***p < 0.01*, ****p < 0.001*, similar to all symbols). Symbols: a vs PPARA-BioCyc, b vs PPARB-BioCyc, c vs PPARG-BioCyc, d vs RXRA, g vs PPARA-PubMed, h vs PPARB-PubMed, j vs RXRA-PubMed, l vs RXRG-PubMed. Non-normal residuals suggest caution in parametric interpretation, though the robust sample size supports validity. (C–E) Natural ligand comparisons demonstrate that indole derivatives exhibit the strongest interactions across all receptors, surpassing short-chain (SCFA), medium-chain (MCFA), long-chain (LCFA), monounsaturated (MUFA), and polyunsaturated fatty acids (PUFA) (*p < 0.001*). PUFA shows intermediate affinity, outperforming LCFA and MUFA (*p < 0.001*) but remaining inferior to indoles. RXR isoforms preferentially bind LCFA, MUFA, and PUFA compared to PPARB and PPARG (*p < 0.001*). Two-way ANOVA followed by Tukey test was performance to detect differences among groups. Boxplots depict median docking scores, 5th–95th percentiles, and significance level are (**p < 0.05*, ***p < 0.01*, ****p < 0.001*, similar to all symbols). Non-normal residuals suggest caution in parametric interpretation, though the robust sample size supports validity. Symbols to C: d vs RXRA, e vs RXRB, f vs RXRG. Symbols to C: D, E: s vs SCFA, p vs PUFA, I vs indoles. Boxplots display median docking scores (central line), 5th–95th percentiles (whiskers), and interquartile ranges (boxes). SCFA: short chain fatty acid; MCFA: medium chain FA; LCFA: long chain FA; MUFA: mono-unsaturated FA; PUFA: poly-unsaturated.

Table S2. Data distribution around median and mean.

	BioCyc					
	**PPARA**	**PPARB**	**PPARG**	**RXRA**	**RXRB**	**RXRG**
Dock metabolites	1039	1031	1036	990	982	979
Brain distribution	119					
GIT High	278					
GIT Low	291					
Minimum	-9.877	-9.034	-10.45	-8.913	-9.383	-9.68
5% percentile	-8.125	-7.047	-7.524	-7.138	-6.851	-6.842
25% percentile	-6.737	-5.763	-5.953	-5.826	-5.558	-5.605
Median	*-5.895*	*-4.947*	*-5.108*	*-4.877*	*-4.578*	*-4.694*
Mean	*-5.749*	*-4.787*	*-5.065*	*-4.786*	*-4.508*	*-4.571*
75% percentile	-4.748	-3.884	-4.086	-3.755	-3.542	-3.541
95% percentile	-3.153	-2.258	-2.759	-2.374	-1.87	-2.072
Maximum	-0.031	1.594	-0.468	1.05	1.469	0.534
	**PubMed**					
	**PPARA**	**PPARB**	**PPARG**	**RXRA**	**RXRB**	**RXRG**
Dock metabolites	171	174	193	163	163	163
Brain distribution	51					
GIT High	69					
GIT Low	29					
Minimum	-9.519	-8.014	-8.566	-7.767	-7.657	-7.283
5% percentile	-8.12	-7.127	-7.169	-7.279	-6.935	-7.004
25% percentile	-6.86	-5.959	-6.083	-6.398	-6.084	-6.212
Median	*-5.818*	*-4.984*	*-5.268*	*-5.632*	*-5.192*	*-5.34*
Mean	*-5.77*	*-4.806*	*-5.042*	*-5.356*	*-5.004*	*-5.161*
75% percentile	-4.795	-3.826	-4.141	-4.621	-4.335	-4.557
95% percentile	-3.161	-2.134	-1.994	-2.402	-1.732	-2.308
Maximum	-0.105	1.378	-0.637	-0.978	-0.755	-0.919

Figure S3A

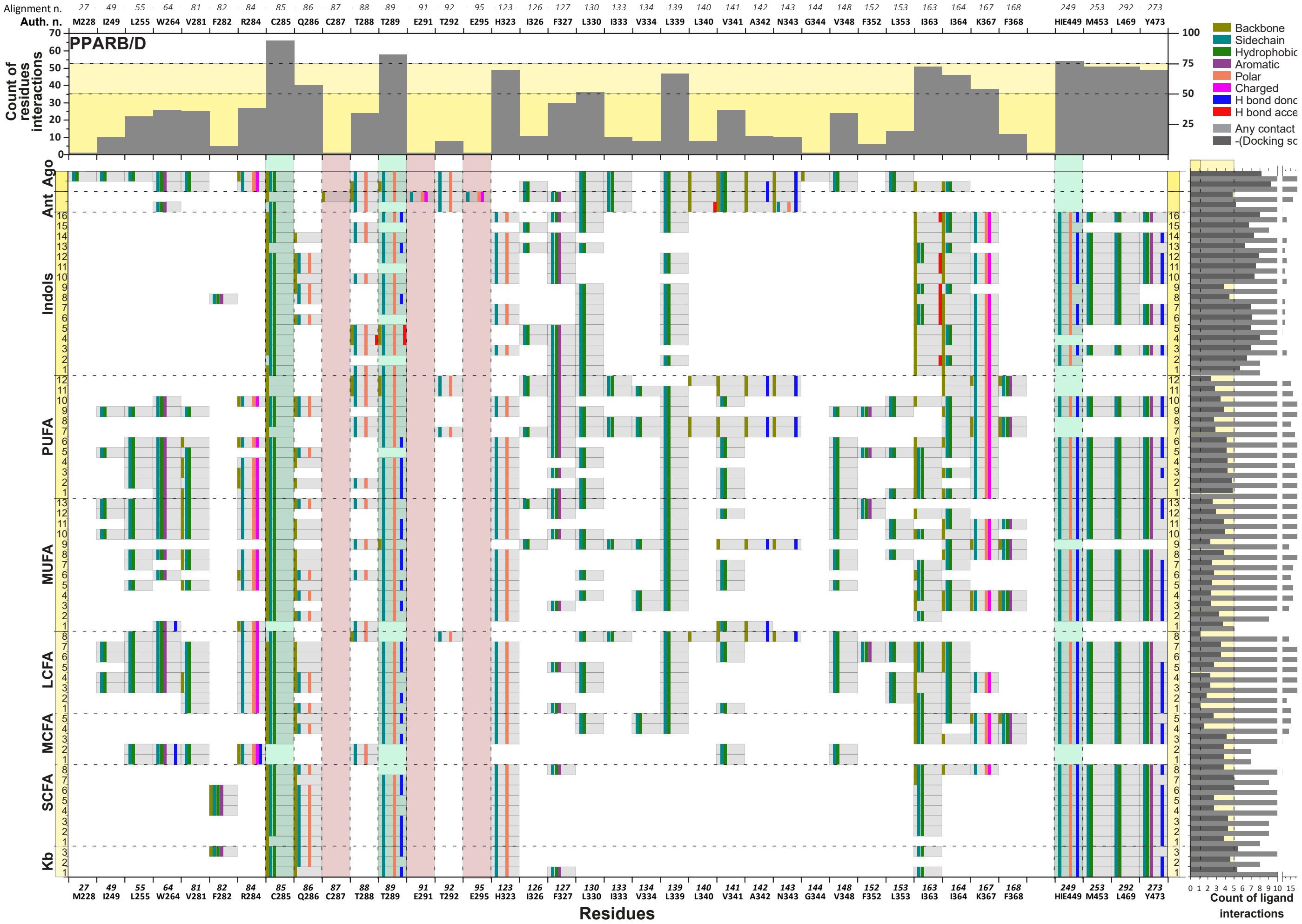

Figure S3B

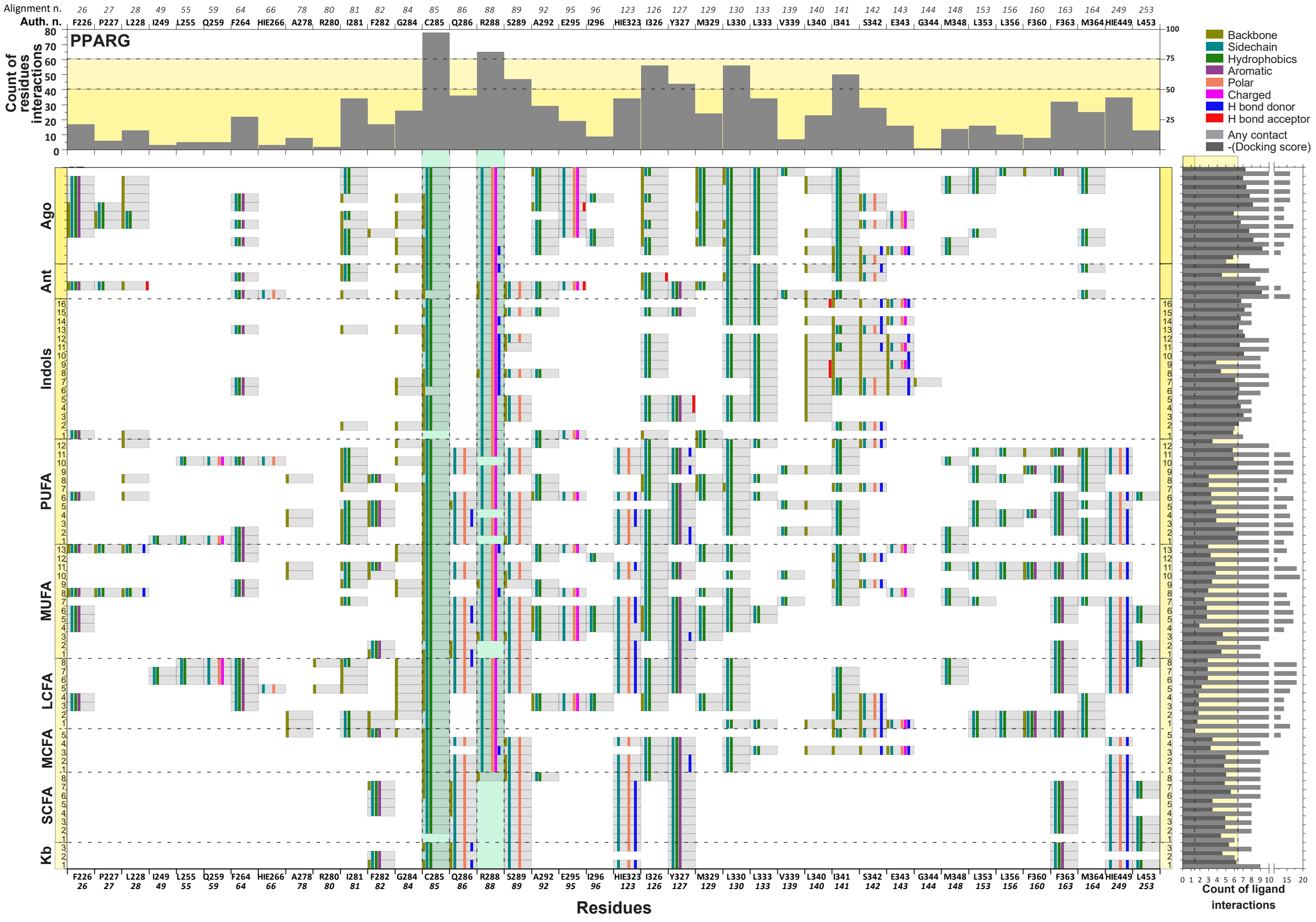

Figure S3C

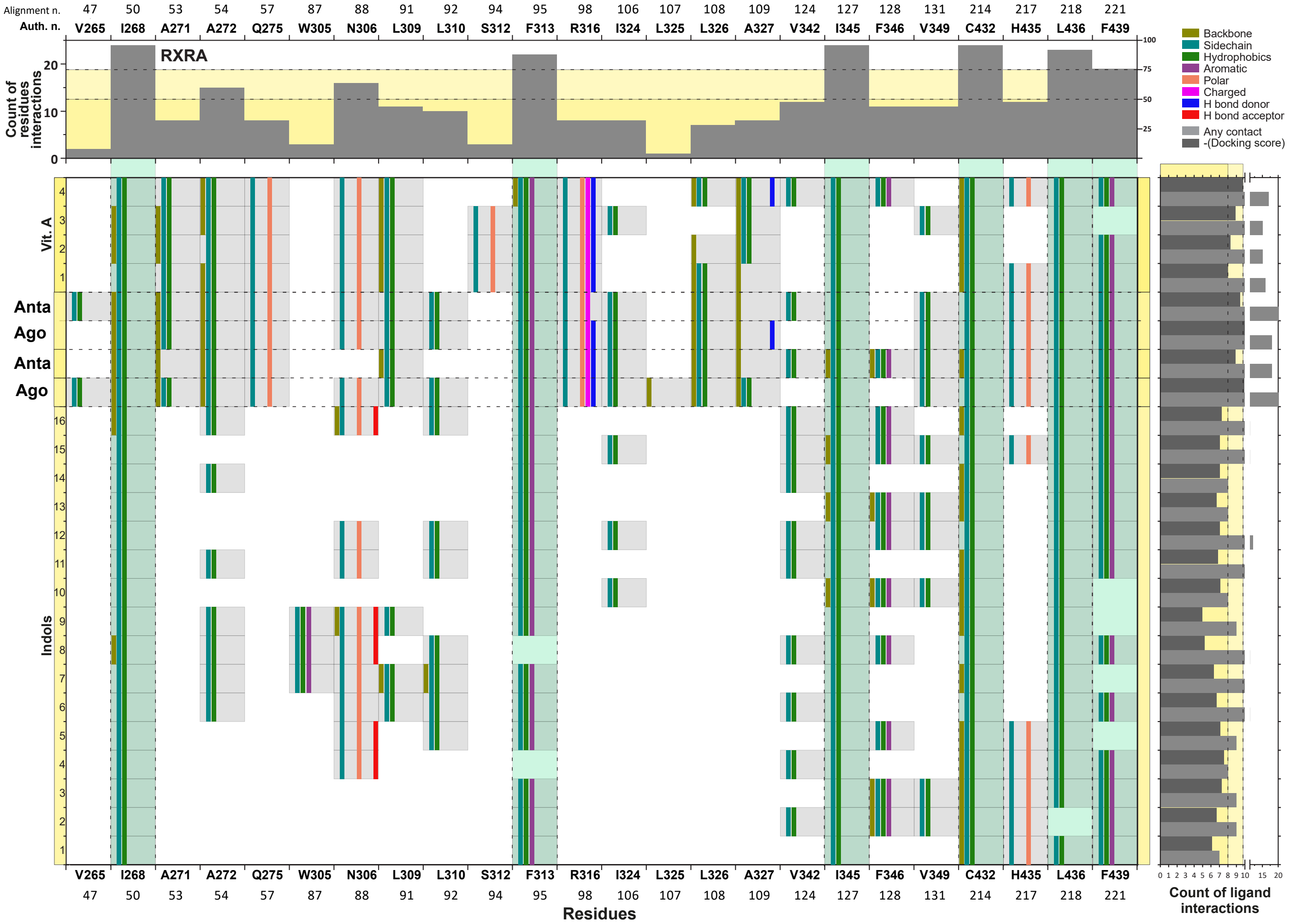

Figure S3D

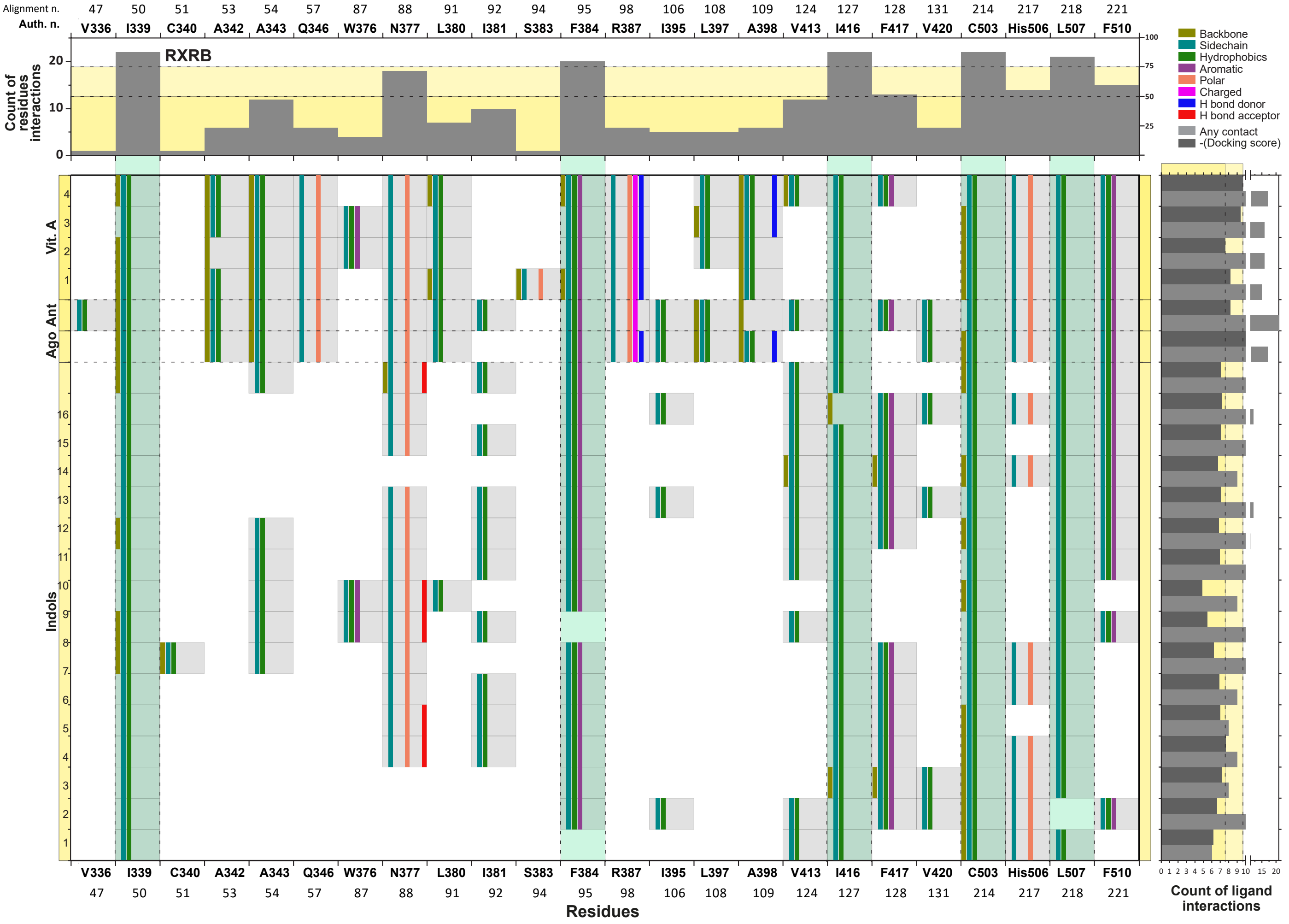

Figure S3E.

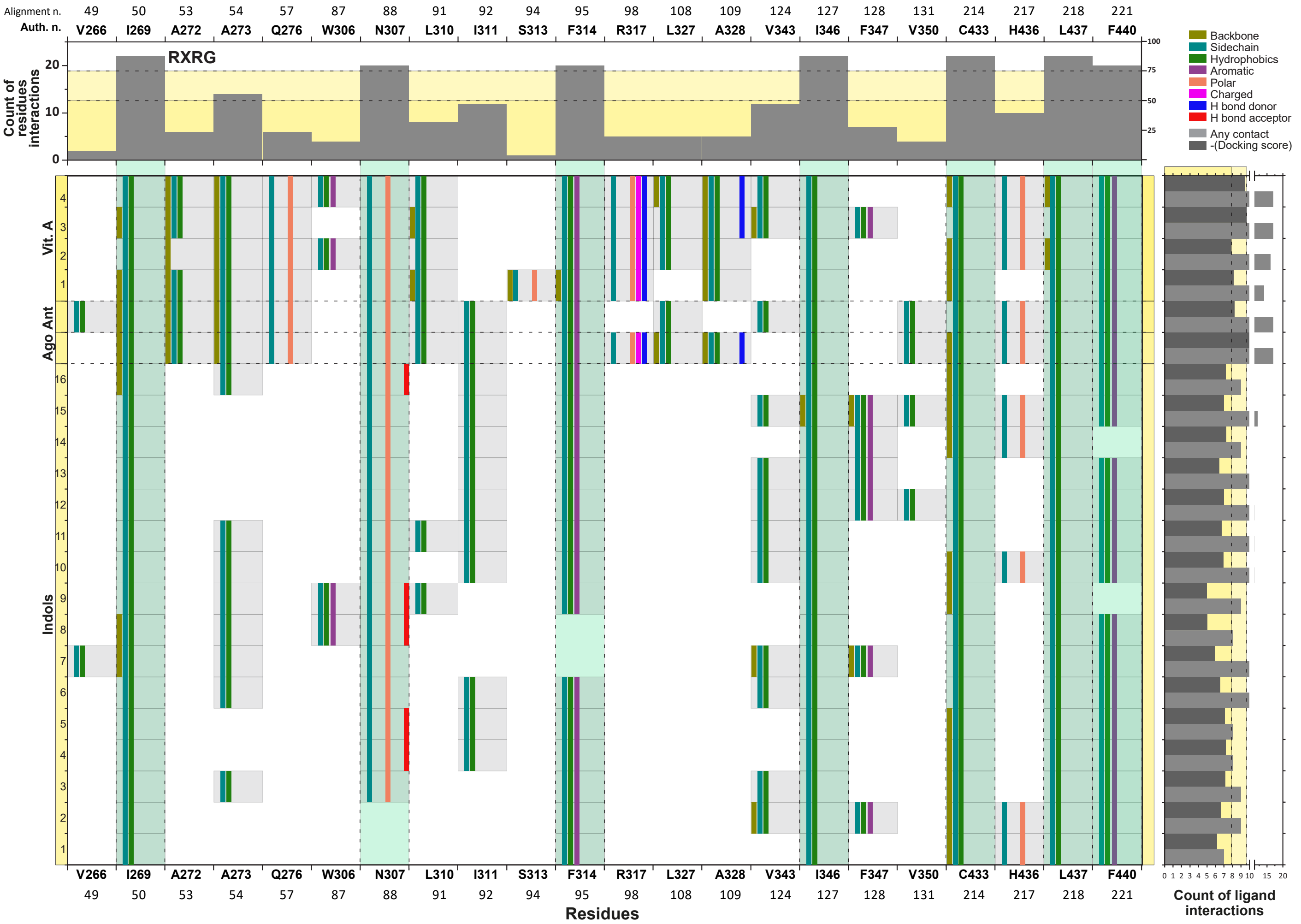

Figure S3. Interaction map or interaction fingerprint of PPARB (A), PPARG (B), RXRA (C), RXRB (D) and RXRG (E) nuclear protein. The map of interactions between nuclear receptor residues (horizontal axis) and natural metabolites, drug reference and indole metabolites are shown (vertical axis). The upper quadrant shows the frequency of any contact (light gray) of the residues with respect to the metabolites and yellow panel and dot line indicate specific residues that interact with ≤50% and ≤75% of total metabolites, respectively. The number of aminoacidic residues are according to alignment and author crystal numbering. The right quadrant shows the frequency of any interactions of the metabolites, with respect to amino acid residues (light gray) and the negative of docking score (dark gray) and dot line and yellow panel indicate maximum and minimum of docking score of reference metabolites, respectively. The main window shows the specific interaction of each amino acid residue and the type of interaction with each of the metabolites. Horizontal dots point the limit of different metabolites or drugs and are indicated by a number relevant to the name in Table 4 and Figure S5. Vertical dots indicate specific aminoacidic residues. Vertical green square indicates specific aminoacidic residue that interacts >75% of metabolites. Vertical red square indicates a specific aminoacidic residue that interacts just with an antagonist. Number of metabolites interactions were color-coded as follows: gray: any interaction; gold: with skeleton; turquoise: with side chain; flag green: with hydrophobic residues; purple: with residues that have aromatic rings; orange: with polar residues; magenta: with charged polar residues; blue: with hydrogen bond donors; red: with hydrogen post acceptors; and white: no interaction. Kb: ketone body; SCFA: short chain fatty acid; MCFA: medium chain FA; LCFA: long chain FA; MUFA: mono-unsaturated FA; PUFA: poly-unsaturated FA; Ant: antagonist; Ago: agonist.

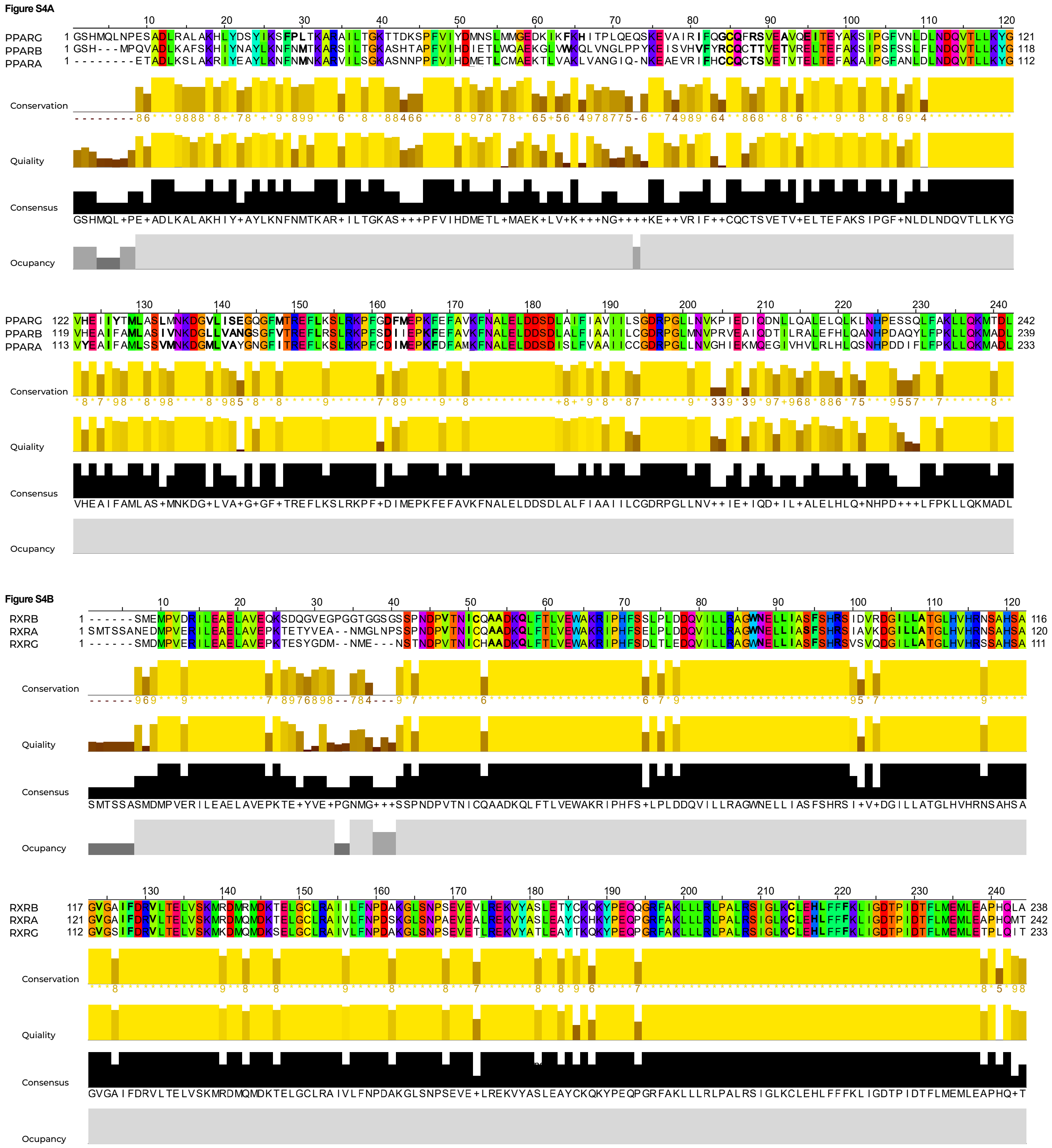

Figure S4. Multiple sequence alignment window of PPRAR (A) and RXR (B) nuclear proteins. Multiple sequence alignment window. The alignment of the protein sequences for the PPAR and RXR nuclear receptors is shown. The alignment was carried out with Clustal Omega (https://www.ebi.ac.uk/jdispatcher/msa/clustalo) and the diagram was generated with the free software Jalviwer 2.11.4.0 to windows (https://www.jalview.org/download/). The color code proposed by (Taylor, 1997) was used for the conserved regions, the quality, the consensus amino acids, and the occupancy.

Table S3. Specific interaction of PPARs

≥75% interaction			
Category	PPARA	PPARB	PPARG
Arm I	C276, Q277, S280, Y314, HIE440, Y464	C285, , T289, HIE449,	C285, R288,
Arm II		, L469	,
Entrance	-	-	-
Charge Clamp		E471, K301	
50-75% interaction			
Category	PPARA	PPARB	PPARG
Arm I	F318, M330, V332	Q286, F327, L330, V341, H323, Y473	Y327, L330, L341, S289, L326, HIE449, I341
Arm II	E251, T253, L254, M355, A256, K257, L258, I339, V444	I363, I364, M453	
Entrance	-	-	-
Charge Clamp	-	-	-
Entrance	<50% interaction		
Category	PPARA	PPARB	PPARG
Arm I	-	-	F363, L453
Arm II	K358, I241, L247, A250, V255, R271, I272, C275, C278, T279, T283, I317, M320, L321, V324, M325, L331, A333, Y334, L344, P359, L456	F352, F282, M228, I249, L255, W264, V281, R284, C287, T288, E291, T292, E295, I326, I333, V334, L339, L340, A342, N343, G344, V348, L353, K367, F368	F226, P227, L228, I249, L255, Q259, F264,HIE266, A278, R280, I281, F282, G284, Q286, A292, E295, I296, HIE323, M329, V339, L340, S342, E343, G344, M348, L353, L356, F360, M364
	N219, M220	-	-
Charge Clamp	-	-	-

Figure S5S.

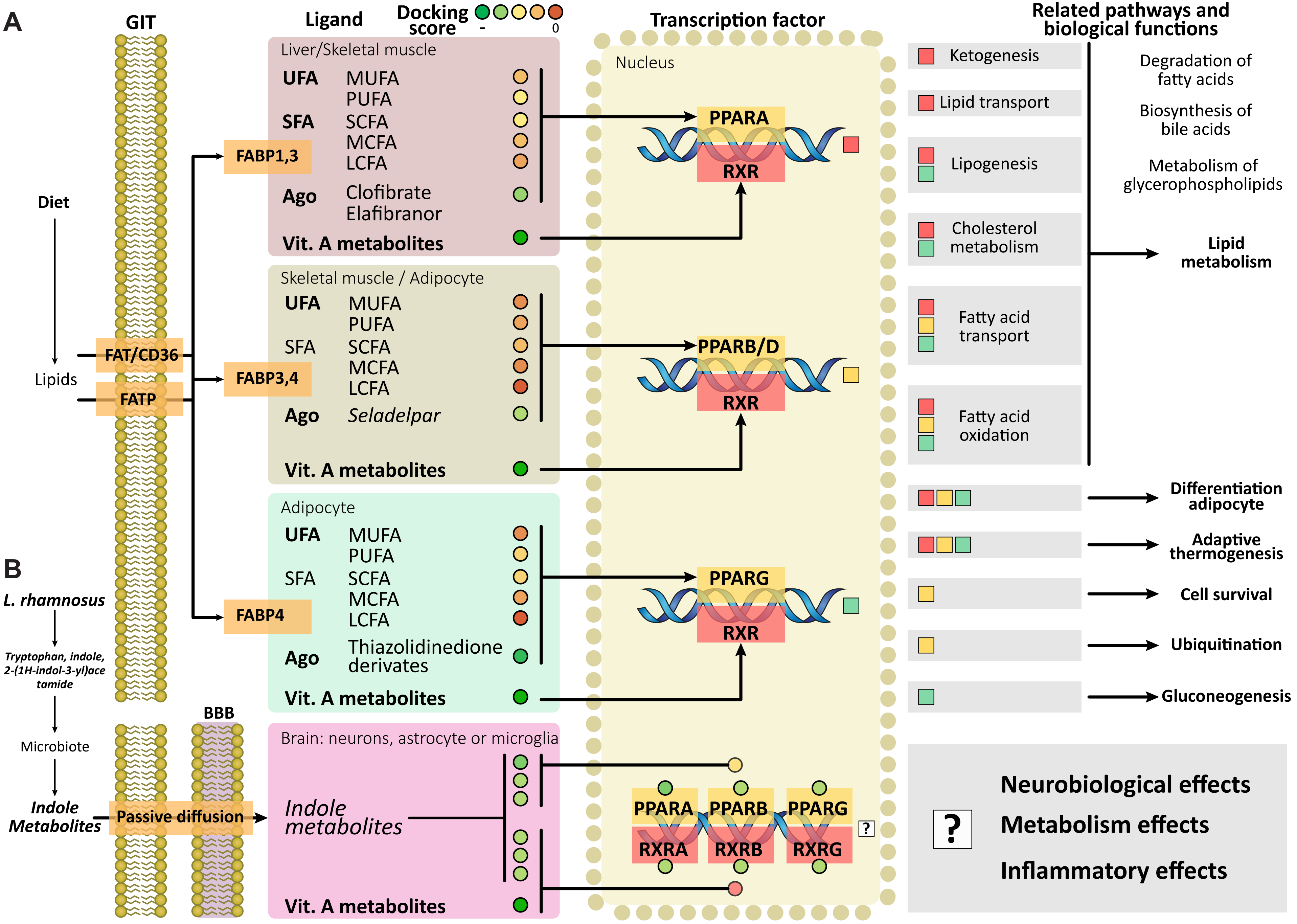
A
GIT
Ligand
Docking score
-
0
Transcription factor
Related pathways and biological functions
Diet
Lipids
FAT/CD36
FATP
FABP1,3
FABP3,4
FABP4
Liver/Skeletal muscle
UFA
MUFA
PUFA
SFA
SCFA
MCFA
LCFA
Ago
Clofibrate
Elafibranor
Vit. A metabolites
Skeletal muscle / Adipocyte
UFA
MUFA
PUFA
SFA
SCFA
MCFA
LCFA
Ago
Seladelpar
Vit. A metabolites
Adipocyte
UFA
MUFA
PUFA
SFA
SCFA
MCFA
LCFA
Ago
Thiazolidinedione derivates
Vit. A metabolites
Nucleus
PPARA
RXR
PPARB/D
RXR
PPARG
RXR
Ketogenesis
Lipid transport
Lipogenesis
Cholesterol metabolism
Fatty acid transport
Fatty acid oxidation
Degradation of fatty acids
Biosynthesis of bile acids
Metabolism of glycerophospholipids
Lipid metabolism
Differentiation adipocyte
Adaptive thermogenesis
Cell survival
Ubiquitination
Gluconeogenesis
B
L. rhamnosus
Tryptophan, indole, 2-(1H-indol-3-yl)ace tamide
Microbiote
Indole Metabolites
Passive diffusion
BBB
Brain: neurons, astrocyte or microglia
Indole metabolites
Vit. A metabolites
PPARA
PPARB
PPARG
RXRA
RXRB
RXRG
?
?
Neurobiological effects
Metabolism effects
Inflammatory effects

Figure S5. Mechanism of action of the PPAR and RXR nuclear receptor pathway and hypothetical mechanism. (A) Image represents mechanisms and biological functions starting with lipid absorption from diet by the GI and distributed to liver, muscle and adipocytes. PPARA plays a role in the clearance of circulating or cellular lipids via the regulation of gene expression involved in lipid metabolism in liver and skeletal muscle. PPARB/D is involved in lipid oxidation and cell proliferation. PPARG promotes adipocyte differentiation to enhance blood glucose uptake. (B) based on our observations we may propose that *L. rhamnosus* normally produces tryptophan, indole and 2-(1H-indol-3-yl)acetamide that could be metabolized by microbiota to produce a cluster of indole metabolites that are distributed to brain by passive diffusion and interact with PPARs and RXRs to produce various neurobiological functions, Metabological effects and Inflammatory effects. Docking score: indicate mean docking score of ligand with respect to PPARs and RXRs. Ago: agonist; Anta: antagonist; BBB: blood brain barrier; FABP4: fatty acid-binding protein adipocyte; FABP1: fatty acid transporter protein, Liver; FABP3: fatty acid transporter protein, Muscle; FATP: fatty acid transporter protein; FAT/CD36: fatty acid translocase/cluster of differentiation 36; GIT: gastrointestinal tract; LCFA: long chain fatty acid; MUFA: mono-unsaturated fatty acid; PUFA: poly-unsaturated fatty acid; SFA: saturated fatty acid; UFA: unsaturated fatty acid; Vit: vitamin. Taken and modified from KEGG DataBase PPAR signaling pathway. Reference pathway map03320 *(https://www.genome.jp/pathway/map03320).*

Table S4. Summary of the genetic characteristics of bacterial species.	*B. animalis lactis BB-12*	*L. rhamnosus GG*
Base pairs	1944152	3010111
Plasmids	0	0
Genes	1601	2775
Genes with molecular function	626	1155
Pseudogenes	18	25
%GC	60.49	46.69
Pathways	138	208
Metabolic Reactions	711	942
Transport	24	115
Transcriptional Units	1204	1841
Publications	71	2078
Total GO	1305	74
Synthesized Compounds	***683***	***923***
Unique Compounds	***171***	***411***
Shared Compounds (Similarity Coefficient)	512	-0.468

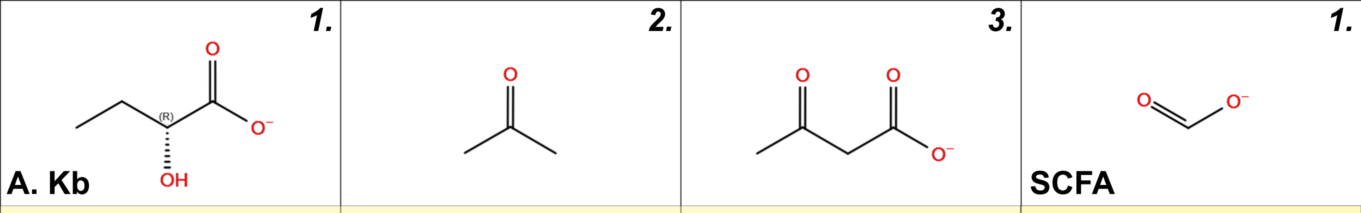

title	4071895	title	180	title	6971017	title	283
PUBCHEM_COMPOUND_CID	4071895	PUBCHEM_COMPOUND_CID	180	PUBCHEM_COMPOUND_CID	6971017	PUBCHEM_COMPOUND_CID	283

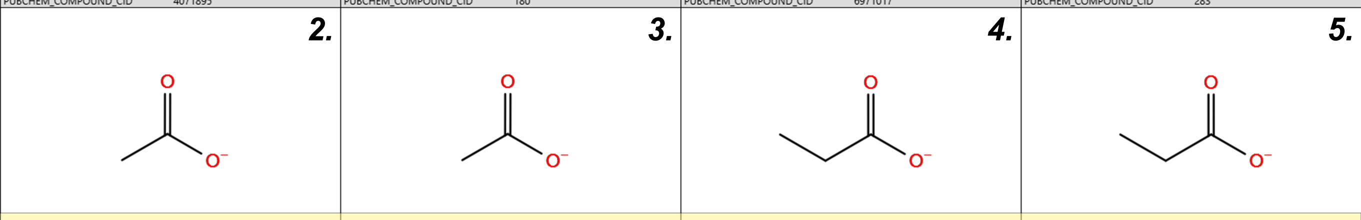

title	175	title	176	title	104745	title	1032
PUBCHEM_COMPOUND_CID	175	PUBCHEM_COMPOUND_CID	176	PUBCHEM_COMPOUND_CID	104745	PUBCHEM_COMPOUND_CID	1032

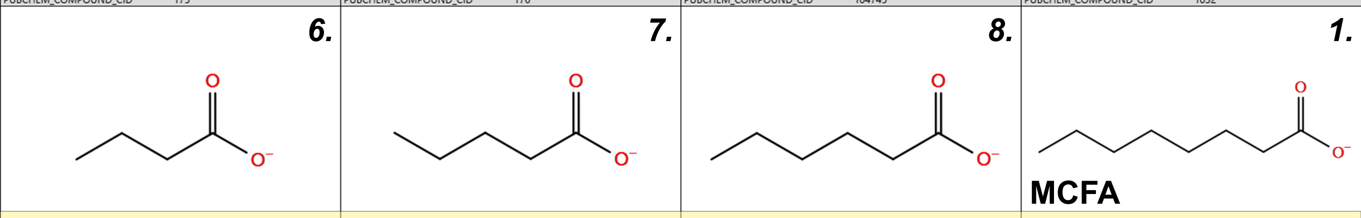

title	264	title	7991	title	8892	title	119389
PUBCHEM_COMPOUND_CID	264	PUBCHEM_COMPOUND_CID	7991	PUBCHEM_COMPOUND_CID	8892	PUBCHEM_COMPOUND_CID	119389

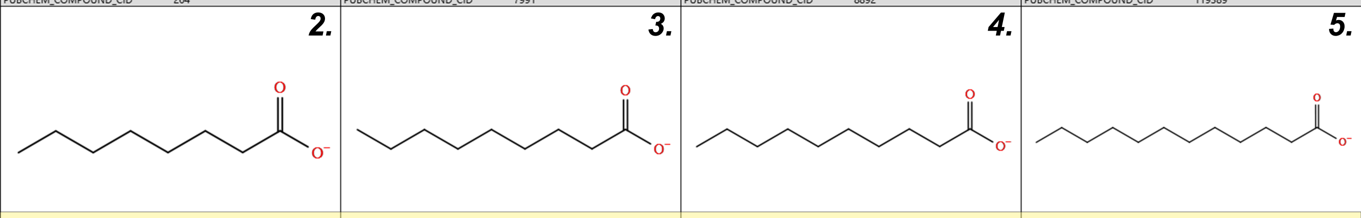

title	379	title	8158	title	2969	title	4149208
PUBCHEM_COMPOUND_CID	379	PUBCHEM_COMPOUND_CID	8158	PUBCHEM_COMPOUND_CID	2969	PUBCHEM_COMPOUND_CID	4149208

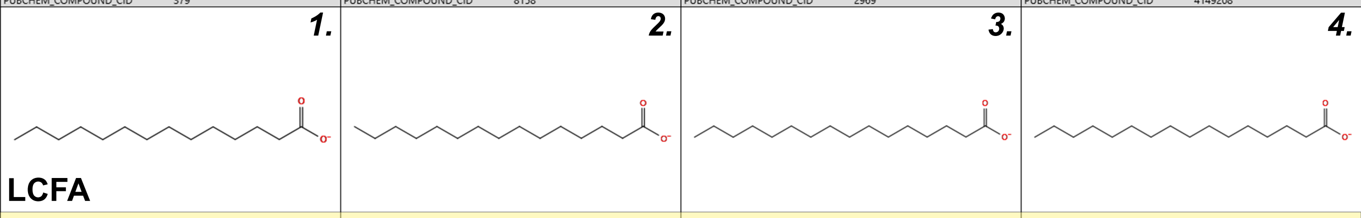

title	11005	title	13849	title	504166	title	985
PUBCHEM_COMPOUND_CID	11005	PUBCHEM_COMPOUND_CID	13849	PUBCHEM_COMPOUND_CID	504166	PUBCHEM_COMPOUND_CID	985

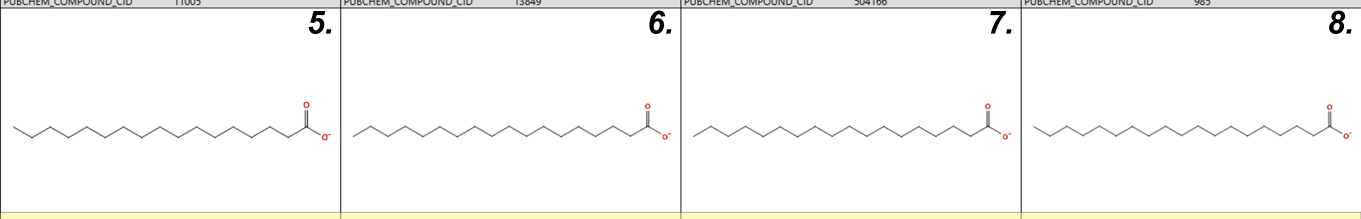

title	10465	title	3033836	title	5281	title	12591
PUBCHEM_COMPOUND_CID	10465	PUBCHEM_COMPOUND_CID	3033836	PUBCHEM_COMPOUND_CID	5281	PUBCHEM_COMPOUND_CID	12591

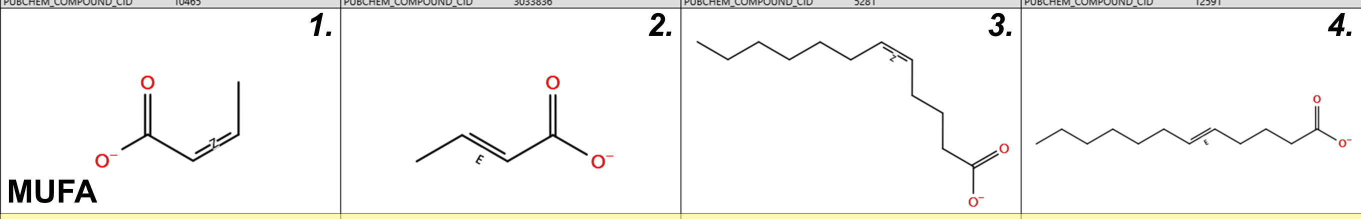

title	19499	title	19499	title	151007 - MUFA - BBB	title	151007 - MUFA - BBB
PUBCHEM_COMPOUND_CID	19499	PUBCHEM_COMPOUND_CID	19499	PUBCHEM_COMPOUND_CID	151007	PUBCHEM_COMPOUND_CID	151007

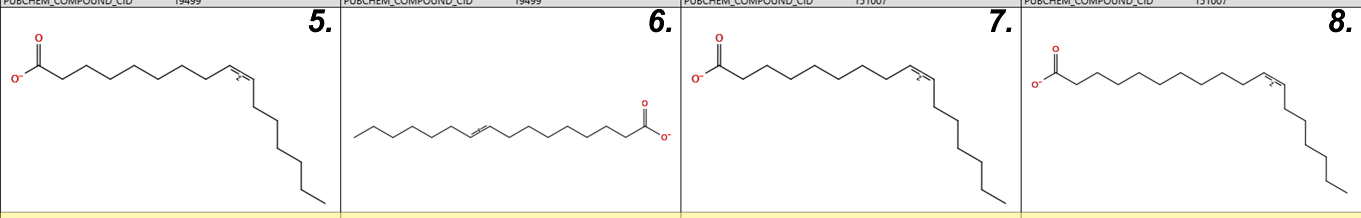

title	5461012 - MUFA - BBB	title	4668	title	4668	title	5461069 - MUFA
PUBCHEM_COMPOUND_CID	5461012	PUBCHEM_COMPOUND_CID	4668	PUBCHEM_COMPOUND_CID	4668	PUBCHEM_COMPOUND_CID	5461069

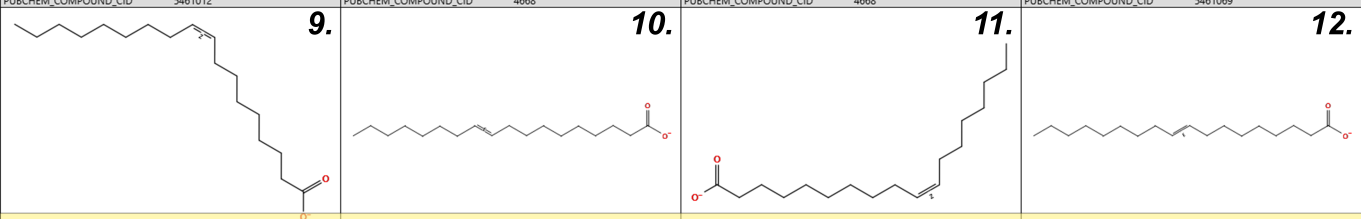

title	5460221 - MUFA - BBB	title	12745	title	12745	title	965
PUBCHEM_COMPOUND_CID	5460221	PUBCHEM_COMPOUND_CID	12745	PUBCHEM_COMPOUND_CID	12745	PUBCHEM_COMPOUND_CID	965

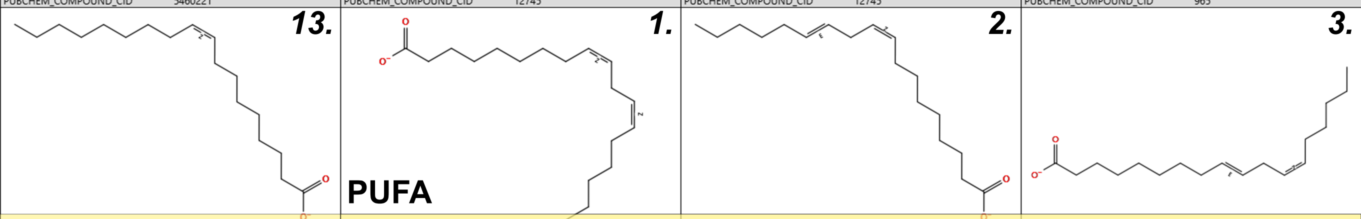

title	965	title	3931 - PUFA - BBB	title	3931 - PUFA - BBB	title	3931 - PUFA - BBB
PUBCHEM_COMPOUND_CID	965	PUBCHEM_COMPOUND_CID	3931	PUBCHEM_COMPOUND_CID	3931	PUBCHEM_COMPOUND_CID	3931

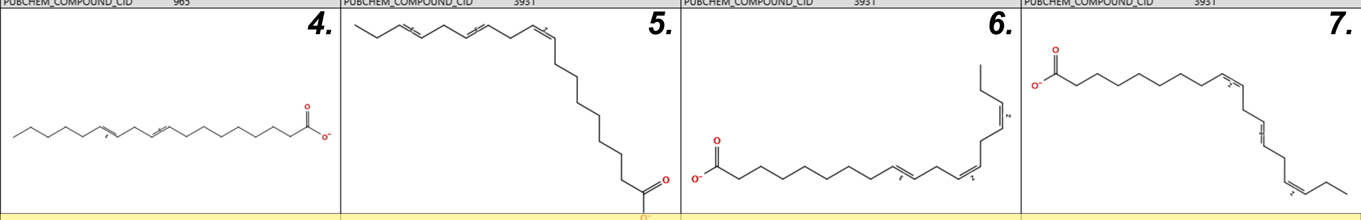

title	3931 - PUFA - BBB	title	860 - PUFA - BBB	title	860 - PUFA - BBB	title	860 - PUFA - BBB
PUBCHEM_COMPOUND_CID	3931	PUBCHEM_COMPOUND_CID	860	PUBCHEM_COMPOUND_CID	860	PUBCHEM_COMPOUND_CID	860

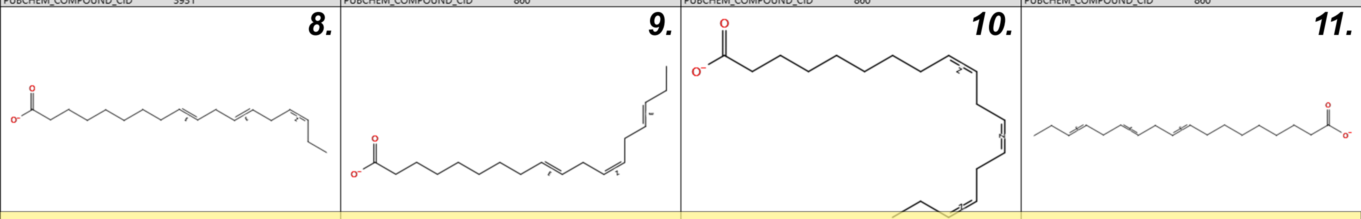

title	860 - PUFA - BBB	title	860 - PUFA - BBB	title	860 - PUFA - BBB	title	860 - PUFA - BBB
PUBCHEM_COMPOUND_CID	860	PUBCHEM_COMPOUND_CID	860	PUBCHEM_COMPOUND_CID	860	PUBCHEM_COMPOUND_CID	860

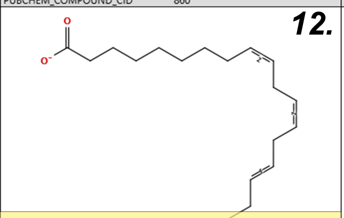

title	860 - PUFA - BBB
PUBCHEM_COMPOUND_CID	860

B. Vit. A

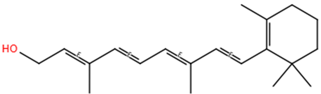

title	445354 - Retinol
PUBCHEM_COMPOUND_CID	445354

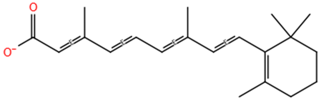

title	6419707 - Retinoate
PUBCHEM_COMPOUND_CID	6419707

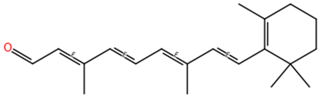

title	63801 - Retinal
PUBCHEM_COMPOUND_CID	638015

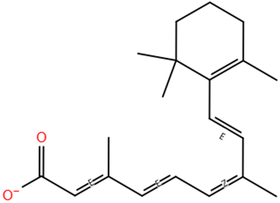

title	449171 - Retinoic acid
PUBCHEM_COMPOUND_CID	449171

C. Indol metabolites

1.

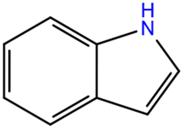

title 798 - Indole - BBB - BBB

2.

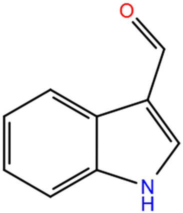

title 10256 - indole - BBB

3.

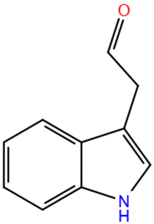

title 800 - Indole - BBB

4.

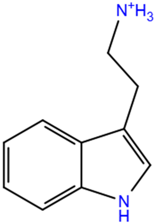

title 1150 - Indole - BBB

5.

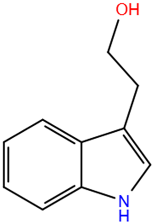

title 10685 - Indole - BBB

6.

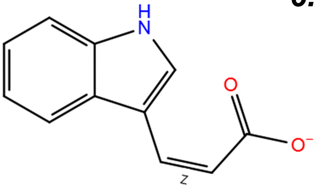

title 14558 - Indole - BBB

7.

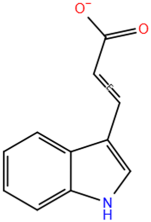

title 14558 - Indole - BBB

8.

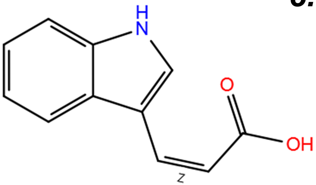

title 14558 - Indole - BBB

9.

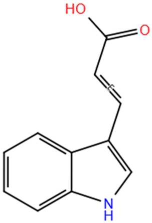

title 14558 - Indole - BBB

10.

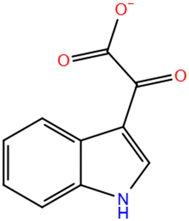

title 73863 - Indole - BBB

11.

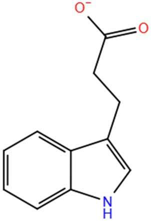

title 3744 - Indole - BBB

12.

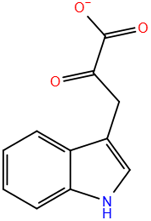

title 803 - Indole - BBB

13.

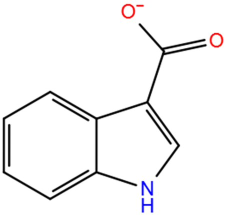

title 6932058 - Indole

14.

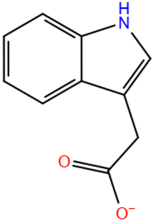

title 801 - Indole

15.

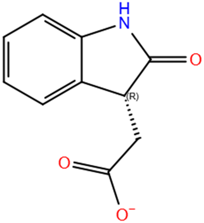

title 3080590 - Indole

16.

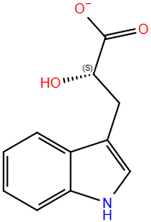

title 92904 - Indole

D. Drugs structures

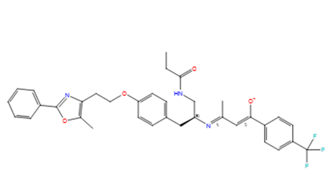

title	446738 - GW 6471- Anta
PUBCHEM_COMPOUND_CID	446738

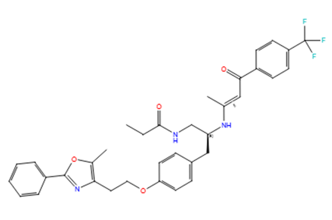

title	446738 - GW 6471- Anta
PUBCHEM_COMPOUND_CID	446738

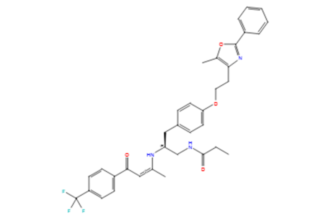

title	446738 - GW 6471- Anta
PUBCHEM_COMPOUND_CID	446738

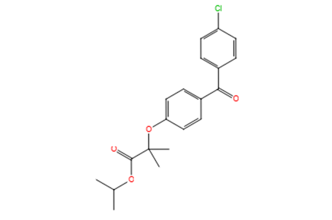

title	3339 - Fenofibrate - Ago
PUBCHEM_COMPOUND_CID	3339

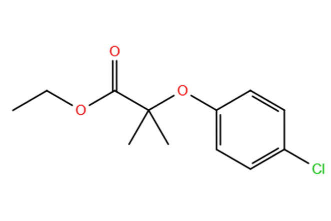

title	2796 - Clofibrate - Ago
PUBCHEM_COMPOUND_CID	2796

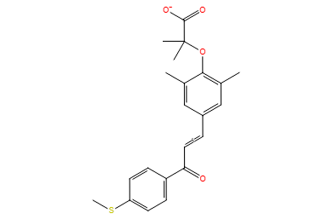

title	9864881 - Elafibranor - Ago
PUBCHEM_COMPOUND_CID	9864881

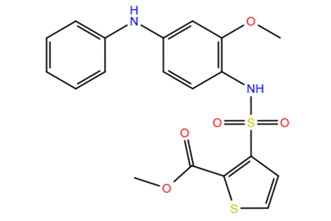

title	46233311 - GSK0660 - Anta
PUBCHEM_COMPOUND_CID	46233311

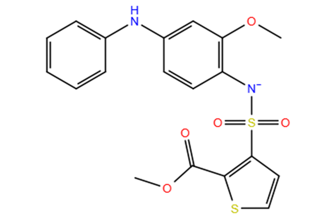

title	46233311 - GSK0660 - Anta
PUBCHEM_COMPOUND_CID	46233311

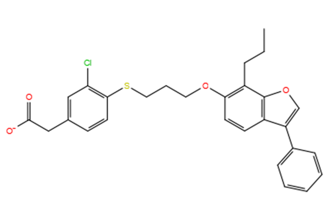

title	9891946 - L-796449 - Ago
PUBCHEM_COMPOUND_CID	9891946

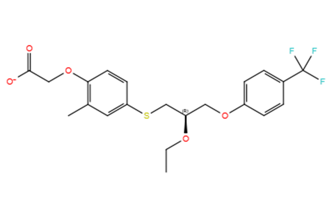

title	11236126 - Seladelpar - Ago
PUBCHEM_COMPOUND_CID	11236126

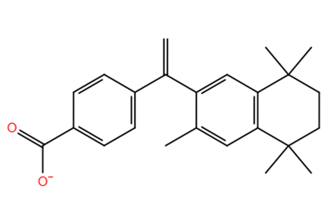

title	82146 - Bexarotene - Anta
PUBCHEM_COMPOUND_CID	82146

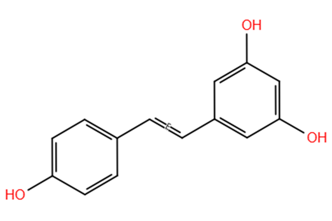

title	445154 - Resveratrol - Anta
PUBCHEM_COMPOUND_CID	445154

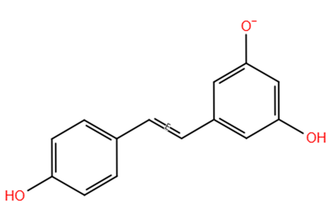

title	445154 - Resveratrol - Anta
PUBCHEM_COMPOUND_CID	445154

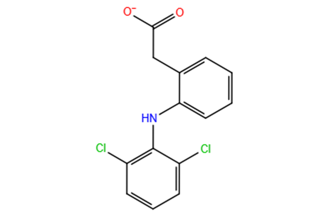

title	3033 - Diclofenac - Anta
PUBCHEM_COMPOUND_CID	3033

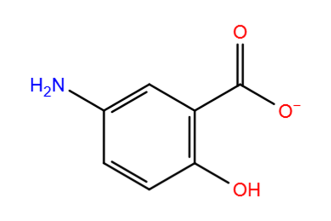

title	4075 - Mesalamine - Ago
PUBCHEM_COMPOUND_CID	4075

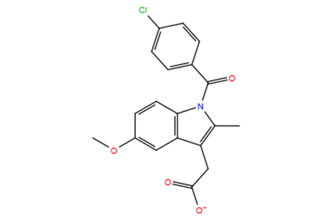

title	3715 - Indomethacin - Ago
PUBCHEM_COMPOUND_CID	3715

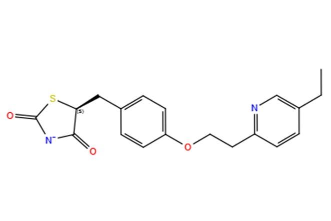

title	4829 - Pioglitazone - Ago
PUBCHEM_COMPOUND_CID	4829

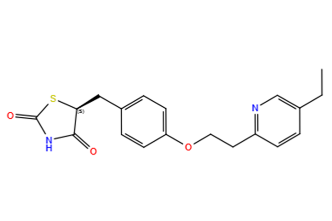

title	4829 - Pioglitazone - Ago
PUBCHEM_COMPOUND_CID	4829

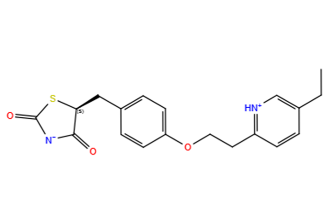

title	4829 - Pioglitazone - Ago
PUBCHEM_COMPOUND_CID	4829

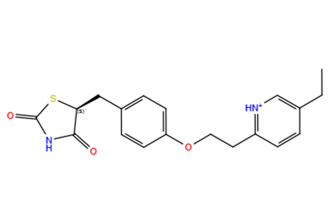

title	4829 - Pioglitazone - Ago
PUBCHEM_COMPOUND_CID	4829

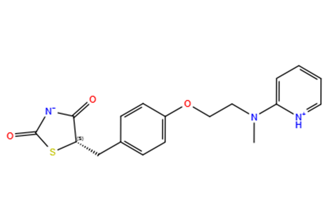

title	77999 - Rosiglitazone - Ago
PUBCHEM_COMPOUND_CID	77999

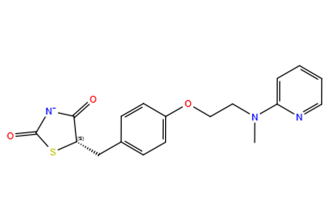

title	77999 - Rosiglitazone - Ago
PUBCHEM_COMPOUND_CID	77999

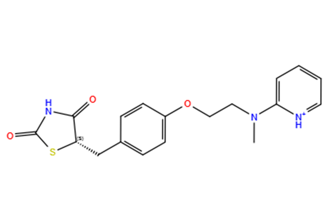

title	77999 - Rosiglitazone - Ago
PUBCHEM_COMPOUND_CID	77999

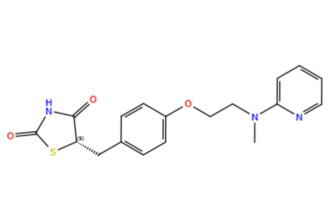

title	77999 - Rosiglitazone - Ago
PUBCHEM_COMPOUND_CID	77999

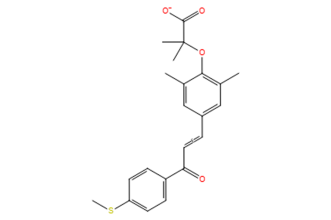

title	9864881 - Elafibranor - Ago
PUBCHEM_COMPOUND_CID	9864881

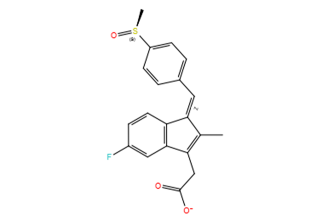

title	1548887 - Sulindac - Anta
PUBCHEM_COMPOUND_CID	1548887

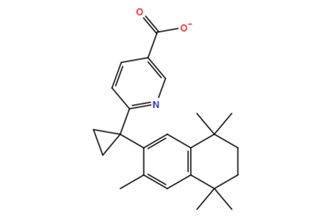

title	3922 - LG-100268 - Anta
PUBCHEM_COMPOUND_CID	3922

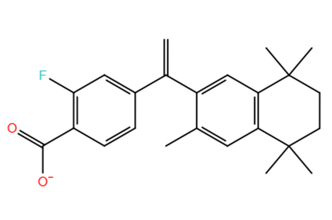

title	25195496 - Fluorobexarotene - Ago
PUBCHEM_COMPOUND_CID	25195496

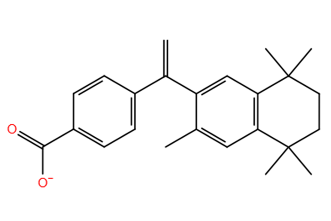

title	82146 - Bexarotene - Activator
PUBCHEM_COMPOUND_CID	82146

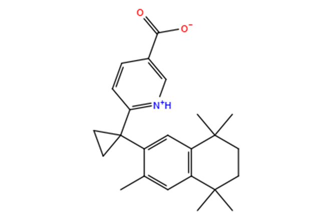

title	3922 - LG-100268 - Anta
PUBCHEM_COMPOUND_CID	3922

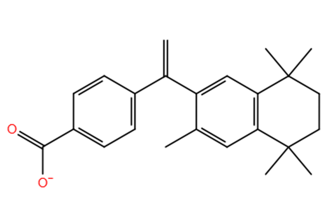

title	82146 - Bexarotene - Activator
PUBCHEM_COMPOUND_CID	82146

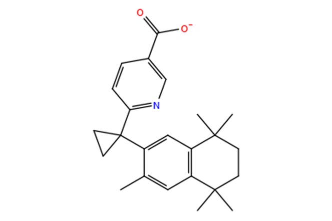

title	3922 - LG-100268 - Anta
PUBCHEM_COMPOUND_CID	3922

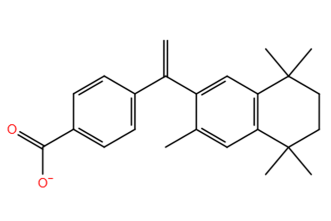

title	82146 - Bexarotene - Activator
PUBCHEM_COMPOUND_CID	82146
